# Global prevalence of psychosocial assessment following hospital-treated self-harm: systematic review and meta-analysis

**DOI:** 10.1192/bjo.2023.625

**Published:** 2024-01-11

**Authors:** Katrina Witt, Katie McGill, Bernard Leckning, Nicole T. M. Hill, Benjamin M. Davies, Jo Robinson, Gregory Carter

**Affiliations:** Centre for Youth Mental Health, The University of Melbourne, Australia; and Orygen, Parkville, Australia; School of Medicine and Public Health, The University of Newcastle, Australia; and Hunter New England Local Health District, Waratah, Australia; Menzies School of Health Research, Charles Darwin University, Australia; School of Population and Global Health, The University of Western Australia, Australia; and Telethon Kids Institute, Nedlands, Australia; Department of Surgery, University of Cambridge, UK; School of Medicine and Public Health, The University of Newcastle, Australia

**Keywords:** Self-harm, suicide, mental health services, psychosocial interventions, risk assessment

## Abstract

**Background:**

Hospital-treated self-harm is common, costly and associated with repeated self-harm and suicide. Providing a comprehensive psychosocial assessment following self-harm is recommended by professional bodies and may improve outcomes.

**Aims:**

To review the provision of psychosocial assessments after hospital-presenting self-harm and the extent to which macro-level factors indicative of service provision explain variability in these estimates.

**Method:**

We searched five electronic databases to 3 January 2023 for studies reporting data on the proportion of patients and/or events that were provided a psychosocial assessment. Pooled weighted prevalence estimates were calculated with the random-effects model. Random-effects meta-regression was used to investigate between-study variability.

**Results:**

119 publications (69 unique samples) were included. Across ages, two-thirds of patients had a psychosocial assessment (0.67, 95% CI 0.58–0.76). The proportion was higher for young people and older adults (0.75, 95% CI 0.36–0.99 and 0.83, 95% CI 0.48–1.00, respectively) compared with adults (0.64, 95% CI 0.54–0.73). For events, around half of all presentations had these assessments across the age range. No macro-level factor explained between-study heterogeneity.

**Conclusions:**

There is room for improvement in the universal provision of psychosocial assessments for self-harm. This represents a missed opportunity to review and tailor aftercare supports for those at risk. Given the marked unexplained heterogeneity between studies, the person- and system-level factors that influence provision of psychosocial assessments after self-harm should be studied further.

Self-harm, which refers to intentional drug overdose, self-injury and/or self-poisoning irrespective of motivation and degree of suicidal intent,^1^ is common, often repeated and strongly associated with suicide.^2^ Hospital-treated self-harm has become a growing public health concern and rates appear to be increasing, particularly in young people, at least according to the number of presentations to general hospitals.^3^ Self-harm also has considerable costs associated with both hospital and aftercare treatment, both in low- to middle-income^4^ and high-income^5^ countries worldwide.

## The importance of providing psychosocial assessments after self-harm

Although effective psychosocial interventions, particularly those based on principles of cognitive–behavioural and dialectical behavioural therapy, have been developed to reduce self-harm repetition in adults,^6^ and to a lesser extent young people,^7^ these interventions are typically delivered as aftercare. Given that many of these treatment approaches are complex, multi-component and relatively prolonged, to review and tailor these aftercare supports professional clinical practice guidelines recommend that all patients presenting to the emergency department of a general hospital following self-harm should also receive a thorough psychosocial assessment. At a minimum, these assessments should thoroughly review the patient's mental health and social needs, circumstances preceding the self-harm act, availability of supports to manage any ongoing or future urges to engage in self-harm, and an assessment of the patient's access to potential lethal means of self-harm.^8,9^

Provision of a comprehensive psychosocial assessment following self-harm is associated with reduced risk of self-harm repetition, particularly among those with no prior contact with psychiatric services.^10^ Routine provision of psychosocial assessments has also been found to promote more frequent discussion of available treatment options, and is associated with a shorter interval between discharge and receipt of follow-up care.^11^ Given their potential cost-effectiveness,^12^ psychosocial assessments are therefore an important component of care.

## Factors affecting provision of psychosocial assessments after self-harm

Several demographic and clinical factors might modify the likelihood that an individual patient is provided with a psychosocial assessment, including age,^13^ gender/sex,^14^ socioeconomic factors,^15^ previous history of psychiatric treatment,^13^ and previous history of self-harm.^13^ At the clinician level, patient-related factors (e.g. method of self-harm, access to social supports), service-level factors (e.g. location, resource limitations owing to high workloads and throughput) and individual staff factors (e.g. confidence, training, knowledge) have all been found to influence decision-making around assessments for people presenting to hospital in suicidal crisis, including following self-harm.^16^ Clinical practices and administrative policies in different settings may also play a role, as might macro-level factors, such as per capita spending on health and availability of trained staff, including specialist mental health professionals.^17^

To date, however, no studies have comprehensively reviewed the proportion of patients receiving psychosocial assessment following an episode of self-harm and the factors that may affect this, with a view to providing recommendations to increase the consistency with which patients are provided these important therapeutic assessments. We therefore undertook a comprehensive review of the international literature to determine the following: (a) the proportion of patients (individuals) and presentations (events) that were provided with a psychosocial assessment after an episode of hospital-treated self-harm; and (b) the factors that affect whether or not psychosocial assessments are provided.

## Method

This review followed guidance in the updated version of the Preferred Reporting Items for Systematic Reviews and Meta-Analyses (PRISMA) statement,^18^ and was pre-registered with the international prospective register of systematic reviews (PROSPERO) database (identifier CRD42021261531). Individual participant consent and ethics approval was not required as only data from previously published studies was included in our analyses.

### Search strategy and selection criteria

The Cochrane Collaboration Depression, Anxiety and Neurosis (CCDAN) specialised register (CENTRAL), Cochrane Database of Systematic Reviews, Medline, EMBASE and PsycINFO were searched from their respective start dates until 3 January 2023, using the search strategy outlined in Supplementary Table 1 available at https://doi.org/10.1192/bjo.2023.625. Nomenclature in this field has changed considerably over the past 60 years. We therefore used keywords designed to capture a diversity of terms and definitions of self-harm. Additionally, the reference lists of identified studies and relevant reviews were hand searched.

All published and unpublished studies reporting data on the proportion of patients (individuals) and/or presentations (events) resulting in a psychosocial assessment after an episode of general hospital-treated self-harm were eligible irrespective of design. Studies were excluded if (a) patients were not recruited or identified from the emergency department of a general hospital, (b) numeric data on the outcome(s) of interest were not reported, (c) data could not be calculated from the information reported, (d) study sample either partially or fully overlapped with that of another included study to ensure data from the same sampling frame was not double counted and (e) published in a language other than English (because of resourcing constraints).

### Data extraction

All records were screened independently by two review authors on title, followed by abstract. Any disagreements were resolved by the senior review author (G.C.). We next retrieved the full texts of studies and pairs of review authors independently screened these full texts, identified studies for inclusion and recorded reasons for exclusion. Once again, disagreements were resolved by discussion with the senior review author (G.C.).

At this stage, we combined multiple publications so that each study, rather than each publication, represented the unit of interest in this review. Where multiple reports of data on the same outcome were reported over the same recruitment period and in the same setting, we preferentially extracted data from the study with the largest denominator (i.e. the primary study) to prevent bias introduced by double counting. Information from secondary studies was only included if data were reported on different outcome(s) or subgroups(s) from the primary study.

For each study, two review authors independently extracted information on (a) study information, including study identifier, year of publication, dates of recruitment and country of publication; (b) participant information, including total number of individuals or events, number of individuals or events lost to follow-up or withdrawn and number of individuals or events analysed; (c) methods, including study design, location and setting; (d) outcomes, including data on the proportion of patients (individuals) and/or admissions (events) provided a psychosocial assessment (numerator and denominator); (e) potential modifying factors (specified *a priori*), including (where possible) mean/median sample age, gender/sex composition, socioeconomic composition, previous history of psychiatric treatment and previous history of self-harm; and (f) notes, including information on study funding and any notable conflicts of interest. Any discrepancies were resolved by a third rater (G.C.). Data extraction commenced on 3 January 2023.

### Outcome measures

The main outcomes of this review were the pooled proportion of patients and/or admissions provided a psychosocial assessment (variously defined, see Supplementary Table 2 for all definitions) within the emergency department following presentation for hospital-treated self-harm. These outcomes could be ascertained using multiple methods, including hospital and/or medical chart review, clinician report, patient self-report, clinical registers or via linkage to population administrative registers.

### Statistical analyses

Quantitative synthesis was performed with the random-effects model.^19^ Accompanying 95% confidence intervals were estimated with the Hartung-Knapp-Sidik-Jonkman adjustment.^20^ We also applied the Freeman–Tukey double-arcsine adjustment, as this does not exclude studies with estimated proportions close to the bounds.^19^ Others, however, have described misleading results in meta-analyses because of problems in the stability of the back-transformation of the Freeman–Tukey double-arcsine adjustment, and recommend use of generalised linear mixed models (GLMM) instead.^21^ Given that GLMM do not enable the calculation of individual study weights,^21^ we preferenced the Freeman–Tukey double-arcsine adjustment in this review, but undertook sensitivity analyses with the GLMM method to investigate any potential impact of transformation choice on the results.

Between-study heterogeneity was assessed with the *I²*-statistic. As high *I²*-values do not necessarily mean data are inconsistent in proportional meta-analyses,^22^ we also report *τ*2 and accompanying 95% confidence intervals. We also explored potential reasons for heterogeneity by investigating whether any of the following potential modifying factors, determined *a priori*, were associated with influencing the prevalence of any of our outcomes, using univariate random-effects meta-regression. These included several macro-level factors: total healthcare spending adjusted to US Dollars, psychiatric beds per 100 000 persons and psychiatrists per 100 000 persons (i.e. potential proxy measures for availability of mental health staffing). These factors were extracted from the World Health Organization Mental Health Atlas within ±5 years of the mid-point of the study recruitment period. We also included several study-level factors, such as study recruitment year (or mid-point in the case of studies that recruited over multiple years), proportion of females, proportion of below average socioeconomic status, proportion with a previous history of psychiatric treatment and proportion with a previous history of self-harm.

Subgroup analyses were next conducted to investigate whether the proportion of individuals and events with each of these outcomes varied by age: young people (mean sample age: ≤25 years), adults (mean sample age: >25 years to <60 years) and older adults (≥60 years). Differences between groups was assessed with the *χ*^2^-test.^19^ Finally, sensitivity analyses using the leave-one-out method was used to investigate the potential influence of each individual study on the pooled estimates. Analyses were undertaken in R software for Windows, version 4.0.5, using the *met*a^23^ and *metafor*^24^ packages.

Publication bias assumes that intervention studies reporting positive results are more likely to be submitted, and subsequently accepted, for publication compared with those reporting negative or null results. However, this assumption may not hold for prevalence studies because there is no consensus as to what the ‘true’ prevalence may be.^19^ Therefore, we followed previous guidance and did not use a statistical test to investigate publication bias.^19^ Instead, we assessed potential publication bias qualitatively.

### Risk-of-bias assessment

Risk of bias was assessed with a tool modified for use with systematic reviews and meta-analyses of prevalence data.^25^ This tool comprises four items affecting external validity (i.e. sample representativeness, sampling frame, participant selection and non-response bias) and seven items affecting internal validity (i.e. data collection, case definition, validity and reliability of outcome assessments, consistency of data collection across cases and controls, appropriateness of observation period, adequacy of the sample size and overall risk of bias). Each item was scored as ‘high’, ‘low’ or ‘unclear’ risk of bias, with the last category indicating either lack of information or uncertainty over the potential for bias. We report a justification for our scores in an accompanying risk-of-bias table, and visualised these judgements with the robvis tool for Windows^26^ in an accompanying figure. Risk-of-bias assessments were conducted by pairs of review authors independently, with any discrepancies resolved by consensus.

## Results

A total of 11 457 records were identified by the electronic search, with three additional records identified following hand searching; 10 890 studies remained eligible for screening following the removal of duplicate records. Following a review of their titles and abstracts, 10 193 were excluded, and a further 480 were excluded following a review of their full texts for the reasons outlined in Supplementary Fig. 1. A further 98 records, representing 79 studies, were excluded from this review, and instead are included in a related review of psychiatric in-/out-patient treatment following self-harm. The interrater reliability between pairs of review authors was moderate (Cohen's *κ* ranged from 0.61 to 0.79).

A total of 119 publications, representing 69 unique samples, were included (see Supplementary Table 2 for the full reference list and methodological details). These 69 studies reported data on 140 021 individual patients and 2 256 706 episodes of self-harm (events). Over half (58.8%) used cross-sectional designs. The included samples had been recruited from 26 different countries. According to World Bank classifications,^27^ most were from high-income countries, including the UK (29 samples), Australia (five samples), the USA (five samples), Finland (two samples), Italy (three samples), South Korea (two samples), Sweden (two samples), Taiwan (two samples), and one each from Belgium, Canada, Denmark, France, Greece, Japan, New Zealand, Norway, Portugal, Qatar, Republic of Ireland and The Netherlands. A number of samples were from upper-middle-income countries, including South Africa (two samples), Turkey (two samples) and Brazil. A smaller number of samples were from lower-middle-income countries, including one each from Nepal, Pakistan and Sri Lanka.

Although most studies included both females and males, in the majority of the 61 samples that reported information on gender/sex composition, over half (59.5%) of the sample were female. Only six of the included samples reported information on socioeconomic level. In these, less than half (34.8%) of the sample were of below average/median socioeconomic status. In the 34 samples that reported information on lifetime history of self-harm, 39.1% had a previously engaged in self-harm before the index episode leading to study inclusion. Finally, in the 26 samples reporting information on lifetime history of psychiatric treatment, just over half (52.6%) had received previous treatment; however, it was not always clear whether this was on an in- and/or out-patient basis.

The weighted mean age of participants at recruitment was 31.6 ± 13.1 years (range: 12.9–74.2 years). On the basis of the average sample age, there were 72 samples that reported data for adults (i.e. those aged >25 years to <60 years; weighted mean age 34.6 ± 3.9 years), 15 that reported data for young people (i.e. those aged ≤25 years; weighted mean age 16.1 ± 3.3 years) and five that reported data for older adults (i.e. those aged ≥60 years; weighted mean age 74.2 ± 0.9 years). Note that some studies contributed data to more than one subgroup if data were available disaggregated by age group.

### Risk of bias

Risk of bias was rated as unclear or high for all included studies, with potential biases most apparent for the domains of representativeness (60 studies), generalisability (32 studies) and acceptability of case ascertainment (50 studies) (see Supplementary Fig. 2 and Supplementary Table 3).

### Proportion of patients (individuals) who had a psychosocial assessment after self-harm

**Fig. 1:**
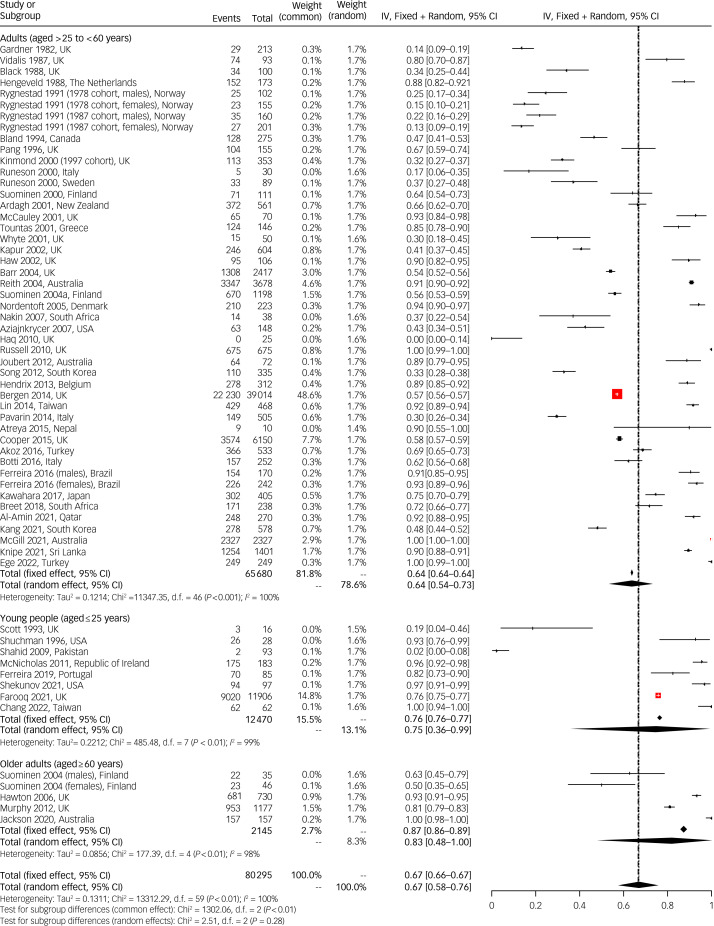
Mixed-effects pooled estimates of the proportion of persons (individuals) resulting in a psychosocial assessment in the emergency department following a hospital presentation for self-harm. The red boxes indicate the weighting applied to the study effect size in the analysis. Please see supplementary material for full reference details of studies mentioned in this figure. IV, inverse variance.

Overall, across the age range nearly two-thirds of patients received a psychosocial assessment in the emergency department following hospital-treated self-harm (random-effects prevalence 0.67, 95% CI 0.58–0.76). Around three-quarters (random-effects prevalence 0.64, 95% CI 0.54–0.73) of adults were provided with these assessments. This compares with three-quarters of young people (random-effects prevalence 0.75, 95% CI 0.36–0.99) and the majority of older adults (random-effects prevalence 0.83, 95% CI 0.48–1.00). Despite this, differences between subgroups were not significant (Fig. 1). As expected, heterogeneity between studies was substantial (*I²* = 99.6%), and the 95% confidence interval around *τ*^2^ excludes zero (*τ*^2^ = 0.13, 95% CI 0.09–0.20), suggesting that some between-study heterogeneity remained unexplained.

### Proportion of presentations (events) that received a psychosocial assessment after self-harm

**Fig. 2:**
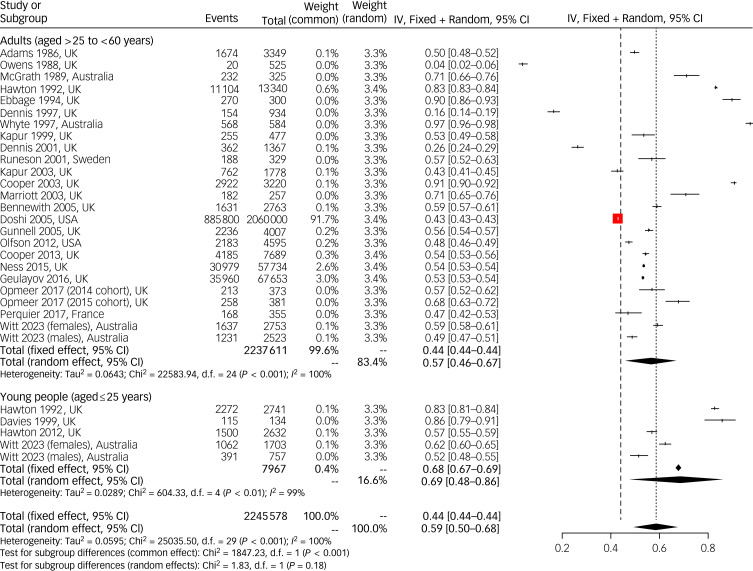
Mixed-effects pooled estimates of the proportion of admissions (events) resulting in a psychosocial assessment in the emergency department following a hospital presentation for self-harm. The red boxes indicate the weighting applied to the study effect size in the analysis. Please see supplementary material for full reference details of studies mentioned in this figure. IV, inverse variance.

With regards to presentations (events), rather than individual patients, just over half of all presentations across the age range received a psychosocial assessment (random-effects prevalence 0.59, 95% CI 0.50–0.68). Half of all presentations by adults received a psychosocial assessment (random-effects prevalence 0.57, 95% CI 0.46–0.67) compared with the majority of presentations by young people (random-effects prevalence 0.69, 95% CI 0.48–0.86). No studies reported data on this outcome for older adults. Again, despite this, differences between subgroups were not significant (Fig. 2). Again, heterogeneity between studies was substantial (*I²* = 99.9%), although the lower bound of the 95% confidence interval for the *τ*^2^ was close to zero (*τ*^2^ = 0.06, 95% CI 0.04–0.11).

### Sensitivity analyses

There was no evidence that transformation choice (i.e. Freedman-Tukey versus GLMM) materially affected the results. Further, influence analyses did not indicate that any one sample was associated with excessive influence.

### Meta-regression

Each one-unit increase in study recruitment year was associated with, on average, a 1.2% increase in the proportion of individuals provided a psychosocial assessment following a hospital presentation for self-harm. There was also some suggestion that a greater proportion of the sample with below average socioeconomic status was associated with a 1.3% reduction in the proportion of individuals receiving a psychosocial assessment following a hospital presentation for self-harm. However, as only six samples reported data on this factor, results should be interpreted with caution (Table 1). No macro-level factor was associated with between-study heterogeneity.

**Table 1:** Univariate random-effects meta-regression effects for macro- and study-level covariates on prevalence estimates

Covariate	Individuals			Events		
	*β*	s.e.	*P-*value	*β*	s.e.	*P-*value
Macro-level covariates						
Total healthcare spending, adjusted to USD (per $1000)	1.56	3.52	0.658	−0.31	0.29	0.288
Psychiatric beds, per 100 000 persons	−0.12	0.09	0.207	−0.01	0.12	0.928
Psychiatrists, per 100 000 persons	−1.78	1.00	0.077	−0.25	1.56	0.874
Study-level covariates						
Study recruitment year	1.23^a^	0.42^a^	0.003^a^	−0.14	0.42	0.730
Percentage of sample, female	0.02	0.23	0.929	0.19	0.21	0.371
Percentage of sample, below average SES	−1.25^a^	0.38^a^	0.001^a^	−	−	−
Percentage of sample, lifetime history self-harm	−0.26	0.42	0.544	0.61	0.44	0.712
Percentage of sample, lifetime history of psychiatric treatment	0.39	0.52	0.456	−0.29	0.40	0.468

Dashes indicate covariates and subgroups with insufficient observations. USD, US Dollar; SES, socioeconomic status.

a. Coefficient is significant at the conventional <0.05 level.

## Discussion

We included 119 publications, representing 69 unique samples, in this review. These samples reported data on 140 021 individual participants and 2 256 706 episodes of self-harm. We found that around two-thirds of adults presenting to emergency departments following an episode of self-harm were provided with a psychosocial assessment. In contrast, three-quarters of young people and almost all older adults were provideed with these assessments. For presentations (events), rather than individual patients, around half of all presentations by adults were provided with a psychosocial assessment, compared with the majority of presentations by young people. No samples reported data for older adults for this outcome. Taken together, results suggest that for adults, repeat episodes of self-harm may be less likely to be provided with a psychosocial assessment, as evidenced by the lower pooled prevalence estimate for presentations (events) compared with individuals (patients). Conversely, for young people, the similar pooled prevalence estimates for individuals (patients) and presentations (events) suggest that repeat self-harm events are usually provided with a psychosocial assessment. The position for older adults is unknown.

More recent studies were associated with an increase in the proportion of individuals provided with a psychosocial assessment following a hospital presentation for self-harm, suggesting some improvements have occurred over time. This may be explained by a number of factors. First, it may be that national clinical practice guidelines, or perhaps more importantly local clinical policy initiatives reflective of these guidelines,^28^ may have resulted in a greater number of self-harm presentations provided with an assessment. Alternatively, improvements in, and uptake of, electronic administrative data systems over time may also account for improved and consistent records of treatment provided within healthcare services, rather than reflecting a genuine change in assessment practice.

Although we found no clear impact of differences in per capita availability of psychiatrists, increasing the number and diversity of professionals capable of providing these assessments through, for example, provision of comprehensive and regular training for all emergency department personnel,^29^ is likely to lead to significant increases in the proportion of presentations receiving these assessments. In particular, given that recent work suggests there is no difference in short-term risks of self-harm repetition by professional background (i.e. psychiatrist versus trained nurses),^30^ providing comprehensive training to nurses to lead these assessments may help to not only increase provision, but also enhance potential scalability to lower-resource settings. Presently, however, only around half of hospital doctors and a quarter of nurses report receiving training in providing psychosocial assessments for self-harm.^31^

Psychosocial assessments should thoroughly review a patient's mental health and social needs, circumstances preceding the self-harm episode, availability of supports to manage any ongoing or future urges to engage in self-harm, and access to potential lethal means of self-harm.^8^ They should also be conducted in a person-centred,^32^ culturally competent,^33^ compassionate and empathetic manner,^34^ and should avoid a formulaic assessment of risk.^32^ The purpose of the assessment should also be made clear at the outset, and where possible, conducted within a private space as soon as practical after presentation.^35^ However, few studies included in this review reported sufficient information on what constituted a psychosocial assessment. Identifying the active component(s) of these assessments should be prioritised to inform the development of future interventions for self-harm.^13^ This is important because some individuals do not experience psychological assessments alone as helpful.^36^

There was marked variability in estimates between studies, as indicated by the very high levels of between-study heterogeneity. It is therefore likely that future, more robust studies may change our confidence in these estimates. Given that none of the macro-level factors investigated by this review were associated with between-study heterogeneity, including psychiatric beds and psychiatrists per 100 000 persons, these findings highlight the likely significant role that local systems of care and context may play in determining how frequently psychosocial assessment for self-harm occurs. Work from the UK, for example, has shown wide variation in the likelihood that psychosocial assessments following self-harm are provided, even within well-resourced settings.^37^ Further research is therefore needed to understand the role that person- and system-level factors may play in influencing provision of psychosocial assessments after hospital-presenting self-harm.

Risk of bias was rated as unclear or high risk of bias for all included studies. Few studies provided data to indicate whether the catchment area was comparable to the relevant national population on important prognostic factors, and therefore, whether prevalence estimates derived from these studies were valid. With regards to generalisability, a number of studies excluded participants either on the basis of self-harm method used, ethnicity and physical and/or psychiatric comorbidities. Finally, with regards to acceptability of case ascertainment, most studies identified self-harm presentations from ICD version 9 or 10 codes alone. However, previous work has demonstrated that the sensitivity of ICD codes in identifying self-harm is low,^38^ and supplementation using textual fields is recommended to improve the enumeration of self-harm where intent is ambiguous.^39^ Improving the quality of future studies reporting the prevalence of psychosocial assessment and other related aspects of the clinical management of self-harm will have an important impact, and may, in fact, change our confidence in the estimates observed in this review.

Finally, according to World Bank classifications,^27^ only 11.6% of samples were recruited from low- to middle-income countries. Previous studies have found that although the 12-month prevalence of self-harm is similar in both low- to middle-income countries and high-income countries,^40^ risks of self-harm repetition may be lower in low- to middle-income countries.^41^ Nevertheless, three-quarters of global suicide deaths occur in these countries.^41^ Given that psychosocial assessments have the potential to offer a relatively low-resource, cost-effective approach,^12^ their development and widescale implementation should be prioritised in these countries as part of national suicide prevention strategies.

In conclusion, routine psychosocial assessment is a critically important part of hospital care for self-harm, particularly because the information gathered will help to engage patients in treatment and inform allocation of aftercare. This review found that around one in three adults, a quarter of young people and almost a fifth of older adults are not provided with a psychosocial assessment following an episode of hospital-treated self-harm. Although there has been modest improvement over time, these effects did not clearly map onto macro-level changes in, for example, per capita availability of psychiatrists or psychiatric beds. Overall, there is considerable scope for improvement both within and between services, to ensure recommendations that all self-harm presentations are provided a comprehensive psychosocial assessment as soon as possible following presentation are met.

## Supporting information

Witt et al. supplementary material Witt et al. supplementary material

## Data Availability

Study data are available from the corresponding author, K.W., on reasonable request.
