# Supplementary material for: Global prevalence of psychosocial assessment following hospital-treated self-harm: systematic review and meta-analysis
Source: BJPsych Open. 2024 Jan 11;10(1):e29. doi: 10.1192/bjo.2023.625 (PMC10790226; doi:10.1192/bjo.2023.625)
Supplement: Witt et al. supplementary material [file S2056472423006257sup001.docx]

**Table SD1.** Systematic search strategy and number of hits current to 3 January 2023.

**Database:**
Embase <1974 to 2022 December 30>
Ovid MEDLINE(R) <1946 to December Week 4 2022>

| ***#*** | **Query** | **Hits** |
| --- | --- | --- |
| 1 | exp Self-Injurious Behavior/ | 104,115 |
| 2 | (overdos* adj3 prevent*).ti,ab | 2,343 |
| 3 | ((nonfatal or non-fatal) adj2 (overdose* or over dose*)).ti,ab | 1,112 |
| 4 | (NSSI* or ((nonsuicid* or non-suicid*) adj2 (self* or injur*))).ti,ab | 4,426 |
| 5 | (suicid* or parasuicid*).ti,ab | 197,138 |
| 6 | (automutilat* or (auto adj mutilat*)).ti,ab | 296 |
| 7 | (selfdestruct* or (self adj destruct*)).ti,ab | 4,481 |
| 8 | (self-harm* or selfharm* or (self adj harm*)).ti,ab | 16,546 |
| 9 | (selfimmolat* or self adjimmolat*).ti,ab | 9 |
| 10 | (selfinflict* or (self adj inflict*)).ti,ab | 5,661 |
| 11 | (selfinjur* or (self adj inflict*)).ti,ab | 5,752 |
| 12 | (selfinjur* or (self adj injur*)).ti,ab | 12,312 |
| 13 | (selfmutilat* or self next mutilat*).ti,ab | 69 |
| 14 | (selfpoison* or (self adj poison*)).ti,ab | 4,387 |
| 15 | (self adj2 (cut or cuts or cutting or cutter* or burn or burns or burning or bite or bites or biting or hit or nits or hitting)).ti,ab | 1,749 |
| 16 | 1 or 2 or 3 or 4 or 5 or 6 or 7 or 8 or 9 or 10 or 11 or 12 or 13 or 14 or 15 | 252,887 |
| 17 | (hospital* or hospital treat*).ti,ab | 3,726,029 |
| 18 | (accident adj2 department).ti,ab | 478 |
| 19 | (emergency adj department).ti,ab | 253,116 |
| 20 | (psychiatr* adj2 (hospital* or ward or unit or $patient)).ti,ab | 52,500 |
| 21 | (mental health adj2 (hospital* or ward or unit or $patient)).ti,ab | 5,046 |
| 22 | 17 or 18 or 19 or 20 or 21 | 3,869,440 |
| 23 | medical* treat*.ti,ab | 138,998 |
| 24 | (refer* or admit* or follow$up or epidemiolog* or prospective* or cohort).ti,ab | 6,766,610 |
| 25 | 23 or 24 | 6,874,825 |
| 26 | 16 and 22 and 25 | 15,381 |
| 28 | limit 27 to english language | 14,033 |

**Figure SD1.** PRISMA flow diagram.


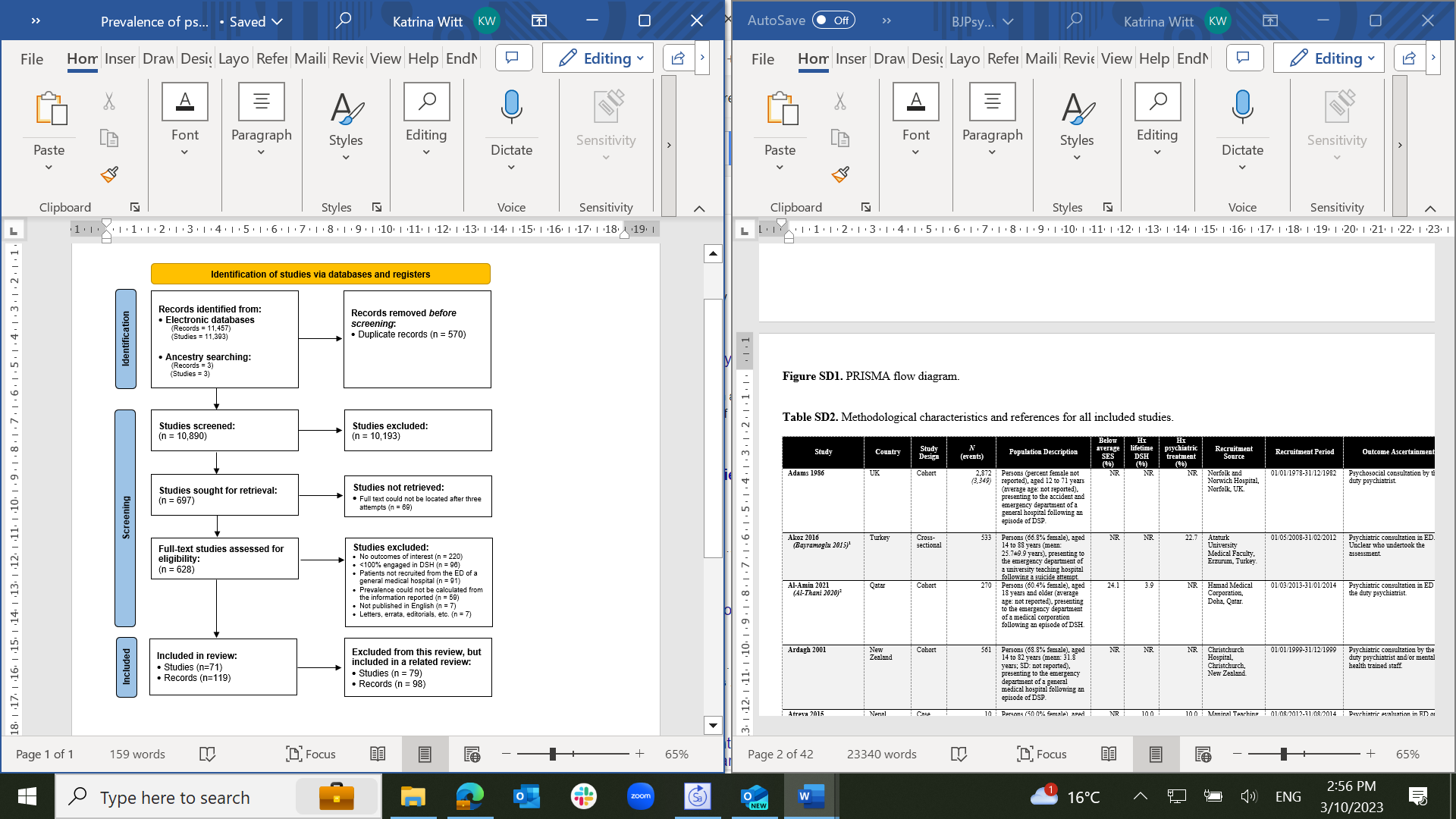


**Table SD2.** Methodological characteristics and references for all included studies.

| **Study** | **Country** | **Study Design** | ***N***  **(events)** | **Population Description** | **Below average SES (%)** | **Hx  lifetime DSH**  **(%)** | **Hx psychiatric treatment**  **(%)** | **Recruitment Source** | **Recruitment Period** | **Outcome Ascertainment** | **Primary Reference** |
| --- | --- | --- | --- | --- | --- | --- | --- | --- | --- | --- | --- |
| **Adams 1986** | UK | Cohort | 2,872  *(3,349)* | Persons (percent female not reported), aged 12 to 71 years (average age: not reported), presenting to the accident and emergency department of a general hospital following an episode of DSP. | NR | NR | NR | Norfolk and Norwich Hospital, Norfolk, UK. | 01/01/1978-31/12/1982 | Psychosocial consultation by the duty psychiatrist. | Adams RHM. (1986). An accident and emergency department’s view of self-poisoning: A retrospective study from the United Norwich Hospitals 1978-1982. *Human Toxicol*, 5: 5-10. |
| **Akoz 2016**  ***(Bayramoglu 2015)*^[[1]](#footnote-1)^** | Turkey | Cross-sectional | 533 | Persons (66.8% female), aged 14 to 88 years (mean: 25.7±9.9 years), presenting to the emergency department of a university teaching hospital following a suicide attempt. | NR | NR | 22.7 | Ataturk University Medical Faculty, Erzurum, Turkey. | 01/05/2008-31/02/2012 | Psychiatric consultation in ED. Unclear who undertook the assessment. | Akoz A, et al. (2016). Can we predict agitation in patients with suicide attempts in the emergency department? *Afri Health Sci*, 16: 831-7. |
| **Al-Amin 2021**  ***(Al-Thani 2020)*^[[2]](#footnote-2)^** | Qatar | Cohort | 270 | Persons (60.4% female), aged 18 years and older (average age: not reported), presenting to the emergency department of a medical corporation following an episode of DSH. | 24.1 | 3.9 | NR | Hamad Medical Corporation, Doha, Qatar. | 01/03/2013-31/01/2014 | Psychiatric consultation in ED by the duty psychiatrist. | Al-Amin H, et al. (2021). Psychosocial and clinical profiles of the cases visiting the emergency department due to acc-idental self-harm and suicide attempts in Doha, Qatar: A retrospective study. *Comm Mental Health J*, 57: 315-24. |
| **Ardagh 2001** | New Zealand | Cohort | 561 | Persons (68.8% female), aged 14 to 82 years (mean: 31.8 years; SD: not reported), presenting to the emergency department of a general medical hospital following an episode of DSP. | NR | NR | NR | Christchurch Hospital, Christchurch, New Zealand. | 01/01/1999-31/12/1999 | Psychiatric consultation by the duty psychiatrist and/or mental health trained staff. | Ardagh M, et al. (2001). Limiting the use of gastrointestinal decontamination does not worsen the outcome from deliberate self-poisoning. *NZ Med J*, 114: 423-5. |
| **Atreya 2015** | Nepal | Case series | 10 | Persons (50.0% female), aged 11 to 73 years (mean: 28.8 years; SD: not reported), presenting to the emergency department of a general hospital following an attempted hanging. | NR | 10.0 | 10.0 | Manipal Teaching Hospital, Pokhara, Nepal. | 01/08/2012-31/08/2014 | Psychiatric evaluation in ED or psychiatric ward. Unclear who undertook the assessment. | Atreya A, et al. (2015). Clinico-epidemiological study of near-hanging cases – an investigation from Nepal. *J Foren Legal Med*, 33: 35-8. |
| **Barr 2004** | UK | Cross-sectional | 2,417 | Persons (52.4% female), aged 16 to 88 years (mean: 34.5±14.1 years), presenting to the emergency department of a general hospital following an episode of DSH. | NR | NR | NR | Conwy and Denbigh-shire NHS Trust, North Wales, UK | 01/01/1996-31/12/2000 | Specialist psychosocial assessment in ED by the duty psychiatrist and/or mental health trained staff. | Barr W, et al. (2004). Self-harm patients who take early discharge from the accident and emergency department: How do they differ from those who stay? *Accident Emerg Nursing*, 12: 108-13. |

| **Bennewith 2005**  ***(Bennewith 2004)*^[[3]](#footnote-3)^** | UK | Cross-sectional | *(2,780)* | Presentations by persons (54.5% female), aged 18 years and older (average age: not reported), presenting to the emergency department of any of 32 general hospitals in the UK following an episode of DSH. | NR | NR | NR | 32 hospitals throughout England, UK | 01/09/2001-30/09/2002 | Specialist psychosocial assessment in ED by mental health trained staff. | Bennewith O, et al. (2005). Factors associated with the non-assessment of self-harm patients attending an Accident and Emergency Department: Results of a national study. *J Affect Disord*, 89: 91-7. |
| --- | --- | --- | --- | --- | --- | --- | --- | --- | --- | --- | --- |
| **Bergen 2014**  ***(Bergen 2007)*^[[4]](#footnote-4)^**  ***(Chang 2015)^[[5]](#footnote-5)^***  ***(Clements 2020)^[[6]](#footnote-6)^***  ***(Diggins 2017)^[[7]](#footnote-7)^***  ***(Gardner 2020)^[[8]](#footnote-8)^***  ***(Farooq 2021)^[[9]](#footnote-9)^***  ***(Haw 2006)*^[[10]](#footnote-10)^**  ***(Hawton 2021)*^[[11]](#footnote-11)^**  ***(Hawton 2007)*^[[12]](#footnote-12)^**  ***(Hawton 2012)*^[[13]](#footnote-13)^**  ***(Hiles 2015)*^[[14]](#footnote-14)^**  ***(Horrocks 2003)*^[[15]](#footnote-15)^**  ***(Kapur 2004)*^[[16]](#footnote-16)^**  ***(Kapur 2006)*^[[17]](#footnote-17)^**  ***(Kapur 2013)*^[[18]](#footnote-18)^**  ***(Kapur 2015)*^[[19]](#footnote-19)^**  ***(Lilley 2008)*^[[20]](#footnote-20)^**  ***(Mahadevan 2010)*^[[21]](#footnote-21)^**  ***(Murphy 2010)*^[[22]](#footnote-22)^**  ***(Murphy 2012)*^[[23]](#footnote-23)^**  ***(Ness 2015)*^[[24]](#footnote-24)^**  ***(Oude Voshaar 2011)*^[[25]](#footnote-25)^**  ***(Pitman 2020)*^[[26]](#footnote-26)^**  ***(Steeg 2018a)*^[[27]](#footnote-27)^**  ***(Steeg 2018b)*^[[28]](#footnote-28)^** | UK | Cohort | 39,014  *(61,583)* | Persons (57.7% female), aged 15 years and older (average age: not reported), presenting to the emergency department of any one of six general medical hospitals following an episode of DSH. Note, data from Farooq (2021) and Hawton (2012) included for analyses relating to children and adolescents, data from Murphy (2012) and Oude Voshaar (2001) included in analyses for older adults only. | NR | NR | NR | Multicentre Study of Self-harm in England, UK. | 01/01/2000-31/12/2010 | Specialist psychosocial assessment in ED by the duty psychiatrist and/or mental health trained staff. | Bergen H, et al. (2014). Alcohol-related mortality following self-harm: A Multi-centre cohort study. *J Roy Soc Med Open*, 5: 1-11. |

| **Black 1988** | UK | Cohort | 100 | Persons (percent female not reported), age range not reported (average age not reported), presenting to the emergency department following an episode of DSP. | NR | NR | NR | Manchester Royal Infirmary, Manchester, UK. | NR | Psychiatric assessment in ED by duty psychiatrist. | Black D, et al. (1988). Assessment of self-poisoning patients by psychiatrists and junior medical staff. *J Roy Soc Med*, 81: 97-9. |
| --- | --- | --- | --- | --- | --- | --- | --- | --- | --- | --- | --- |
| **Bland 1994** | Canada | Cross-sectional | 275 | Persons (61.8% female), aged 12 to 80 years (average age: not reported), who presented to the emergency department of a general hospital following an episode of DSH. | NR | 41.0 | NR | University of Alberta Hospital, Edmonton, Canada. | 01/11/1990-30/05/1991 | Assessment by the duty psychiatrist in ED. | Bland RC, et al. (1994). The epidemic-ology of parasuicide in Edmonton. *Can J Psychiatry*, 39: 391-6. |
| **Botti 2016** | Italy | Cross-sectional | 252 | Persons (38.5% female), aged 16 to 78 years (mean: 42.9 years; SD: not reported), presenting to the emergency department of a general hospital and then admitted to a specialist toxicology unit following an episode of DSP as per local protocols. | NR | NR | NR | Florence Careggi University Hospital, Florence, Italy | 01/01/2010-31/12/2014 | Psychiatric evaluation in toxicology unit. Unclear who undertook the assessment. | Botti P, et al. (2016). Toxic love: A 3-year retrospective analysis of love-related toxicological deliberate self-harm. *Clin Toxicol*, 54: 461. |
| **Breet 2018** | South Africa | Cross-sectional | 238 | Persons (59.7% female), aged 16 to 46 years (mean: 31.5±13.8 years), presenting to the emergency department of a general hospital following an episode of DSH. | 55.0 | 45.8 | NR | Hospitals within the Western Cape Province, South Africa | 16/06/2014-29/03/2015 | Psychiatric assessment in ED. Unclear who undertook the assessment. | Breet E, et al. (2018). Chronic sub-stance use and self-harm in a primary health care setting. *Afr J Prm health Care Fam Med*, 10: a1544. |
| **Chang 2022** | Taiwan | Cross-sectional | 62  *(74)* | Children and adolescents (66.1% female), aged under 18 years (median: 16.1 years, IQR:9.2-18.8 years), present-ing to the emergency depart-ment of one of two general hospitals following an episode of DSH. | 12.9 | 38.7 | NR | Taipei Tzu Chi Hospital and Chi Mei Medical Center, Taiwan. | 01/01/2016-31/12/2019 | Psychiatric assessment in ED. Unclear who undertook the assessment. | Chang T-H, Yu C-H, Yiang G-T, et al. (2022). Characteristics of child-ren and adolescents presenting to the emergency department with self-inflicted injury: Retrospective  analysis of two teaching hospitals. *Pediatr Neonatol*, 63: 131-8. |
| **Cooper 2003** | UK | Cross-sectional | *(3,220)* | Presentations by persons (57.0% female), aged 11 to 87 years (mean: 31.0 years), presenting to the emergency department of one of three general hospitals following an episode of DSH. | NR | 62.1 | 56.2 | Manchester and Salford Self-harm Project, Manchester, UK | 01/09/1997-31/08/1999 | Clinical risk assessment in ED by the duty doctor (unclear if psych-iatrist and/or if mental health trained). | Cooper JB, et al. (2003). Factors that influence emergency department doctors’ assessment of suicide risk in deliberate self-harm patients. *Eur J Emerg Med*, 10: 283-7. |
| **Cooper 2015**  ***(Cooper 2013)*^[[29]](#footnote-29)^** | UK | Cohort | 6,347  *(7,689)* | Persons (55.6% female), aged 18 to 94 years (average age not reported), presenting to any one of 32 hospitals in England, UK following an episode of DSH. Note that data from Cooper 2015 relates to individuals whilst data from Cooper 2013 relates to events. | NR | 51.0 | 10.0 | 32 hospitals throughout England, UK | 01/05/2010-30/06/2011 | Psychosocial assessment in ED by the duty psychiatrist and/or mental health trained staff. | Cooper J, et al. (2015). Variations in the hospital management of self-harm and patient outcome: A multi-site observational study in England. *J Affect Disord*, 174: 101-5. |
| **Davies 1999** | UK | Cross-sectional | 124  *(134)* | Children and adolescents (85.5% female), aged 11 to 16 years (mean: 14.6 years; SD: not reported), admitted to the emergency department of a general hospital following an episode of attempted suicide. | NR | 34.8 | NR | Altnagelvin Area Hospital, London-derry, Northern Ireland, UK | 01/01/1995-30/04/1997 | Psychiatric assessment by the duty psychiatrist. | Davies M, et al. (1999). Adolescent parasuicide in the Foyle area. *Ir J Psych Med*, 16: 9-12. |
| **Dennis 1997** | UK | Cross-sectional | 854  *(934)* | Persons (51.5% female), aged 16 to 90 years (mean: 32.0±14.2 years), presenting to the accident and emergency department of a general hospital following an episode of DSH. | NR | 47.5 | 66.7 | Leicester Royal Infirmary, Leicestershire, UK | 01/04/1994-31/03/1995 | Psychiatric consultation in ED by duty psychiatrist and/or mental health trained staff. | Dennis M, et al. (1997). An exam-ination of the accident and emergency management of deliberate self harm. *J Accid Emerg Med*, 14: 311-5. |
| **Dennis 2001** | UK | Cross-sectional | *(1,359)* | Presentations by persons (percent female not reported), aged 16 years and older (average age: not reported), presenting to the emergency department of a general medical hospital following an episode of DSH. Note, only attendances related to DSH were included in this study. | NR | 72.3 | 96.1 | Leicester Royal Infirmary, Leicestershire, UK | 01/10/1997-30/09/1998 | Psychosocial assessment by mental health trained staff. | Dennis M, et al. (2001). The psycho-social assessment of deliberate self harm: Using clinical audit to improve the quality of the service. *Emerg Med J*, 18: 448-50. |
| **Doshi 2005** | USA | Cross-sectional | *(2,060,000)* | Presentations by persons (57.2% female), of any age (mean: 31.0 years; SD: not reported; range: not reported), presenting to any one of a number of emergency departments of general hospitals located throughout the USA. Note that "...[t]he number of participating EDs differed each year, with 392 in 1997, 398 in 1998, 376 in 1999, 376 in 2000, and 397 in 2001" (p.370). | NR | NR | NR | National Hospital Ambulatory Medical Care Survey, USA | 01/01/1997-31/12/2001 | Mental status examination in ED. Unclear who undertook the assessment. | Doshi A, et al. (2005). National study of US emergency department visits for attempted suicide and self-inflicted injury, 1997-2001. *Ann Emerg Med*, 46: 369-75. |
| **Ebbage 1994** | UK | Cross-sectional | *(300)* | Presentations by persons (54.0% female), aged 10 years and older (average age: not reported), presenting to the emergency departments of one of two general hospitals following an episode of DSP. | NR | NR | NR | St Bartholomew's Hospital, Royal London Hospital, Newham Hospital, Whipps Cross Hospital, North East London, UK | NR | Psychosocial history and psych-ological assessmentin ED by duty physician and/or on-call psych-iatrist. | Ebbage J, et al. (1994). The psycho-social assessment of patients discharged from accident and emergency departments after deliberate self-poisoning. *J Roy Soc Med*, 87: 515-6. |
| **Ege 2022** | Turkey | Cohort | 249 | Persons (65.5% female), aged 20-43 years (mean: 32.3±12.1 years), presenting to the emergency service of a general hospital following a suicide attempt. | NR | 19.7 | NR | NR | 01/01/2015-31/12/2017 | Psychiatric consultation in ED. Unclear who undertook the assessment. | Ege D, Türkdoğan KA, Memİş ÇO, et al. (2022). Decision-making in suicidal attempt to discharge from  emergency department or to consult psychiatry: A retrospective study.  *Turk Kinikleri J Med Sci*, 42: 1-4. |

| **Ferreira 2016** | Brazil | Cohort | 412 | Persons (58.7% female), aged 12 to 83 years (mean: 32.6±14.3 years), admitted to the emergency unit of an academic teaching hospital following an episode of DSH. Note, data disaggregated by gender/sex group. | 32.0 | NR | NR | Ribeirão Preto Medical School, University of Sao Paulo, Brazil | 01/01/2006-31/12/2007 | Psychiatric assessment in ED by the duty psychiatrist. | Ferreira AD, et al. (2016). Clinical features, psychiatric assessment, and longitudinal outcome of suicide attempters admitted to a tertiary emerg-ency hospital. *Arch Suicide Res*, 20: 191-204. |
| --- | --- | --- | --- | --- | --- | --- | --- | --- | --- | --- | --- |
| **Ferreira 2019** | Portugal | Case series | 85  *(90)* | Children and adolescents (83.5% female), aged 12 to 17 years (mean: 15.0 years; SD: not reported), admitted to the emergency department of a general hospital (level II) following an episode of intentional drug overdose. | NR | NR | 63.0 | NR | 01/01/2014-31/12/2018 | Psychiatric evaluation in ED. Unclear who undertook the assessment. | Ferreira AB, et al. (2019). Intentional drug intoxication in adolescents: Who, when, how and why? *Eur J Pediatr*, S27. |
| **Gardner 1982** | UK | Cross-sectional | 213 | Persons (percent female not reported), aged 14 years and older (average age: not reported), presenting to the emergency department of a general hospital following an episode of DSP. | NR | NR | NR | Addenbrookes Hospital, Cambridge, UK | 01/11/1978-31/05/1979 | Assessment in ED by consultant or sernior registrar psychiatrists. | Gardner R, et al. (1982). Psychological and social evaluation in cases of deliberate self-poisoning seen in an accident department. *Br Med J*, 284: 491-3. |
| **Geulayov 2016** | UK | Cross-sectional | 47,023  *(84.353)* | Persons (56.8% female), aged 15 to 97 years (mean: 32.1±14.0 years), presenting to the emergency department of any one of six general medical hospitals following an episode of DSH. | NR | NR | 62.3 | Multicentre Study of Self-harm in England, UK. | 01/01/2012-31/12/2012 | Specialist psychosocial assessment in ED by the duty psychiatrist and/or mental health trained staff. | Geulayov G, et al. (2016). Epidemi-ology and trends in non-fatal self-harm in three centres in England, 2000-2012: Findings from the Multicentre Study of Self-harm in England. *BMJ Open*, 6: e010538. |
| **Gunnell 2005** | UK | Cross-sectional | *(4,033)* | Presentations by persons (54.8% female), aged 18 to 95 years (median: 33.0 years), presenting to the emergency department of one of 31 general hospitals following an episode of DSH. | NR | NR | NR | Various hospitals, England, UK | 01/09/2001-30/09/2002 | Specialist psychosocial assessment in ED by mental health trained staff. | Gunnell D, et al. (2005). The epidemic-ology and management of self-harm amongst adults in England. *J Public Health*, 27: 67-73. |
| **Haq 2010** | UK | Cross-sectional | 25 | Persons (68.0% female), average age not reported, range not reported, presenting to the emergency department of a general medical hospital following an episode of DSH. | NR | NR | 64.0 | NR | 01/02/2009-31/08/2009 | Psychiatric assessment with full mental state examination in ED by staff without specialist mental health training and/or experience. | Haq SU, et al. (2010). Assessment of self harm in an accident and emergency service – the development of a proforma to assess suicide intent and mental state in those presenting to the emergency department with self harm. *Psychiatr Danub*, 22(suppl. 1): S26-32. |
| **Haw 2002** | UK | Cohort | 106 | Persons (61.3% female), aged 15 years and older (average: not reported; range: not reported), presenting to the emergency department of a general medical hospital following an episode of DSH and who were diagnosed with depressive disorder as per ICD-10 research criteria. | NR | 68.9 | NR | Oxford Monitoring System, Oxford, UK | 10/02/1997-01/12/1997 | Assessment by the duty psychiatrist. Setting not stated. | Haw C, et al. (2002). Deliberate self harm patients with depressive disorders: Treatment and outcome. *J Affect Disord*, 70: 57-65. |

| **Hawton 1992a**  ***(Hawton 1990)*^[[30]](#footnote-30)^**  ***(Hawton 1992b)*^[[31]](#footnote-31)^**  ***(Hawton 1996)*^[[32]](#footnote-32)^**  ***(Hawton 2006)*^[[33]](#footnote-33)^** | UK | Cohort | 9,605  *(13,340)* | Persons (62.1% female), aged 15 years and older (average age: not reported), presenting to a general teaching hospital following an episode of DSH. Data from Hawton 1992b and 1996 included in analyses for children and adolescents, data from Hawton 2006 included in analyses for older persons. | NR | 30.5 | NR | Oxford Monitoring System, Oxford, UK | 01/01/1976-31/12/1990 | Specialist psychosocial assessment in ED by the duty psychiatrist, and/or mental health trained staff. | Hawton K, et al. (1992). Trends in deliberate self poisoning and self injury in Oxford, 1976-90. |
| --- | --- | --- | --- | --- | --- | --- | --- | --- | --- | --- | --- |
| **Hendrix 2013** | Belgium | Cross-sectional | 312 | Persons (60.9% female), aged 16 to 86 years (median: 35.0 years), presenting to the emergency department of an academic teaching hospital following an episode of DSP. | NR | 31.8 | 54.0 | University Hospitals Leuven, Leuven, Belgium | 01/01/2009-31/12/2009 | Assessment by the duty psych-iatrist in ED. | Hendrix L, et al. (2013). Deliber-ate self-poisoning: Characteristics of patients and impact on the emergency depart-ment of a large university hospital. *Emerg Med J*, 30: e9. |
| **Hengeveld 1988** | The Netherlands | Cross-sectional | 173  *(189)* | Persons (62.4% female), aged 12 to 79 years (mean: 36.0 years; SD: not reported), presenting to a general medical hospital following a suicide attempt. | NR | NR | 24.0 | University Hospital Leiden, Leiden, The Netherlands | 01/02/1983-30/09/1983 | Assessment by the duty psych-iatrist. Setting not stated. | Hengeveld MW, et al. (1988). Evaluation of psychiatric consul-tations with suicide attempters. *Acta Psychiatr Scand*, 77: 283-9. |
| **Joubert 2012** | Australia | Cross-sectional | 72  *(94)* | Persons (50.0% female), average age not reported, range not reported, presenting to a general medical hospital following a suicide attempt. | NR | NR | NR | Western (Footscray) Hospital, Melbourne, Australia | 12/12/2000-31/01/2001 | Psychiatric assessment in ED by the duty psychiatrist and/or mental health trained staff. | Joubert L, et al. (2012). Suicide attempt presentations at the emergency depart-ment: Outcomes from a pilot study examining precipitating factors in deliberate self-harm and issues in primary care physician management. *Soc Work Health Care*, 51: 66-76. |
| **Kang 2021**  ***(Cho 2020)*^[[34]](#footnote-34)^** | South Korea | Cross-sectional | 578 | Persons (65.4% female), aged 19 to 56 years (mean: 38.6±19.1 years), presenting to the emergency department of a general hospital following an episode of DSH. | 52.8 | 42.3 | NR | Inje University Sanggye Paik Hospital, Seoul, South Korea | 01/03/2017-31/12/2019 | Psychiatric consultation. Unclear who undertook the assessment. | Kang M, et al. (2021). Unique charac-teristics that distinguish suicide attempt-ers from patients with nonsuicidal self-injury admitted to the emergency department following self-harm behaviour: Psychological scales and biochemical makers. *Turk J Emerg Med*, 21: 62-8. |

| **Kapur 2003**  ***(Kapur 2002)*^[[35]](#footnote-35)^** | UK | Cohort | 604  *(1,778)* | Persons (54.4% female), aged 16 years and older (mean: 33.1 years; SD: not reported), presenting to the emergency departments of one of six hospitals (three teaching hospitals and three district hospitals) following DSP. Note, data from Kapur 2002 included in analyses for individuals, data from Kapur 2003 included in analyses for events. | NR | 38.6 | NR | 6 hospitals, North-West England, UK | NR | Psychosocial assessment in ED. Unclear who undertook the assessment. | Kapur N, et al. (2003). Service pro-vision and outcome for deliberate self-poisoning in adults: Results from a six centre descriptive study. *Soc Psychiatry Psychiatr Epidemiol*, 38: 390-5. |
| --- | --- | --- | --- | --- | --- | --- | --- | --- | --- | --- | --- |
| **Kapur 1999** | UK | Cohort | 458  *(477)* | Persons (48.7% female), aged 16 years and older (mean: 30.9±11.8 years; range: not reported), presenting to the emergency department of any one of four teaching hospitals following an episode of DSP. | NR | 16.8 | NR | Multicentre Study of Self-harm in England, UK | 18/11/1996-16/12/1996 | Psychosocial assessment by the duty psychiatrist and/or mental health trained staff. | Kapur N, et al. (1999). General hospital services for deliberate self-poisoning: An expensive road to nowhere? *Postgrad Med J*, 75: 599-602. |
| **Kawahara 2017** | Japan | Cohort | 405  *(499)* | Persons (70.1% female), aged 12 to 88 years (mean: 39.7±15.8 years), admitted to the emergency department of a general medical centre following an episode of DSH. | NR | 54.6 | 76.3 | National Hospital Organization Kumam-oto Medical Center Kumamoto, Japan | 01/04/2013-31/03/2014 | Psychiatric consultation in ED by the duty psychiatrist. | Kawahara YY, et al. (2017). Predictors of short-term repetition of self-harm among patients admitted to an emer-gency room following self-harm: A retrospective one-year cohort study. *Psychiatry Res*, 258: 421-6. |
| **Kinmond 2000** | UK | Cross-sectional | 645 | Persons (54.3% female), average age not reported, range not reported, presenting to the emergency department of a general hospital following an episode of DSH. Note, data relevant to this review reported for the 1997 cohort only. | NR | NR | NR | NR | 1990 cohort:  01/01/1990-30/06/1990  1997 cohort:  01/01/1997-30/06/1997 | Referred for psychiatric opinion and psychosocial assessment in ED. Unclear who undertook the assessment. | Kinmond K, et al. (2000). Attendance for self-harm in a West Midlands hospital A&E department. *Br J Nursing*, 9: 215-20. |
| **Knipe 2021** | Sri Lanka | Cross-sectional | 1,401 | Persons (54.3% female), aged 18 to 38 years (average age not reported), presenting to the emergency department of a tertiary care teaching hospital following an episode of DSP. | NR | NR | NR | Teaching Hospital Peradeniya, Pera-deniya, Sri Lanka. | 01/01/2019-31/08/2020 | Psychiatric assessment in the ED undertaken by either a medical officer (42.3% of cases), a registrar (34.9% of cases), a senior registrar (5.4% of cases), or a consultant (4.7% of cases). In 7.8% of cases it was unclear who undertook the assessment. | Knipe D, Silva T, Aroos A, et al. (2021). Hospital presentations for self-poisoning during COVID-19 in Sri Lanka: An interrupted time-series analysis. *Lancet Psychiatry*, 8: 892-900. |
| **Lin 2014** | Taiwan | Cross-sectional | 468 | Persons (80.1% female), age range not reported (mean age 33.6±12.3 years), presenting to the emergency department of a general hospital following an episode of DSH. | NR | NR | NR | Mackay Memorial Hospital, Taipei, Taiwan | 01/06/2004-30/05/2006 | Psychosocial assessment in ED by psychiatrist and/or social workers. | Lin C-J, et al. (2014). The character-istics, management, and aftercare of patients with suicide attempts who attended the emergency department of a general hospital in northern Taiwan. *J Chinese Med Assoc*, 77: 317-24. |

| **Marriott 2003** | UK | Cross-sectional | *(266)* | Presentations by persons (53.8% female), aged 12 years or older (average: not reported), presenting to the emergency department of one of two general hospitals following an episode of DSH. | NR | 72.3 | 62.1 | Leeds General Infirmary and Leeds Teaching Hospital, West Yorkshire, UK | NR | Psychosocial assessment by the duty psychiatrist and/or mental health trained staff. | Marriott R, et al. (2003). Assessment and management of self-harm in older adults attending accident and emerg-ency: A comparative cross-sectional study. *Int J Geriatr Psychiatry*, 18: 645-52. |
| --- | --- | --- | --- | --- | --- | --- | --- | --- | --- | --- | --- |
| **McCauley 2001** | UK | Cross-sectional | 70 | Persons (62.9% female), aged 17 to 32 years (mean: 32.1±14.7 years), admitted to a rural general hospital following an episode of DSH. | NR | 47.7 | 81.5 | NR | 01/01/1997-31/12/1998 | Psychosocial assessment by members of psychiatric team. Setting not stated. | McCauley MD, et al. (2001). Assess-ment following deliberate self-harm: Who are we seeing and are we follow-ing the guidelines? *Ir J Psych Med*, 18: 116-9. |
| **McGill 2021**  ***(Dani 2022)*^[[36]](#footnote-36)^**  ***(McGill 2022)*^[[37]](#footnote-37)^**  ***(Jackson 2020)*^[[38]](#footnote-38)^** | Australia | Cohort | 2,327 | Persons (62.3% female), aged 18 years and older (average: not reported), admitted to a general medical hospital following an episode of DSP.  Note, data from Dani (2022) only included for analyses relating to young people, data from Jackson (2020) included only for analyses related to older adults, and data from McGill (2022) only included for the 2017 cohort. | NR | NR | 64.6 | Hunter Area Toxicology Service, Newcastle, Australia | 01/07/2003-30/06/2013 | Mental health/psychiatric assessment by the duty psychiatrist. | McGill K, et al. (2021). Comparison of accredited person and medical officer discharge decisions under the Mental Health Act of NSW: A cohort study of deliberate self-poisoning patients. *Aust N Z J Psychiatry*. ePub ahead of print, DOI: 10.1177/00048674211009613. |
| **McGrath 1989** | Australia | Cross-sectional | 300  *(325)* | Persons (60.6% female), aged 12 to 78 years (mean: 30.7±13.0 years), admitted to the emergency department of a general hospital following an episode of DSP. | NR | 46.8 | 71.4 | Princess Alexandra Hospital, Brisbane, Australia | 01/10/1985-31/09/1986 [sic] | Psychiatric consultation. Unclear who undertook the assessment. | McGrath J. (1989). A survey of deliberate self-poisoning. *Med J Aust*, 150: 317-24. |
| **McNicholas 2011** | Republic of Ireland | Cross-sectional | 183 | Children and adolescents (75.9% female), aged 6 to 17 years (average: not reported), presenting to a general paediatric hospital following an episode of DSH. | NR | 29.0 | NR | Our Lady’s Children’s Hospital Crumlin, County Dublin, Republic of Ireland | 01/01/1993-31/12/2003 | Psychiatric assessment in ED. Unclear who undertook the assessment. | McNicholas F, et al. (2011). Deliberate self-harm in children and adolescents: An 11-year case note study. *Ir J Psych Med*, 28: 191-5. |
| **Nakin 2007** | South Africa | Cross-sectional | 73 | Persons (62.7% female), aged 20 to 40 years (average: not reported), admitted to the emergency department of one of two general hospitals following a suicide attempt. | NR | 12.0 | NR | NR | 01/06/2001-31/03/2002 | Psychiatric assessment in ED. Unclear who undertook the assessment. | Nakin DCT, et al. (2007). Evaluation of attempted-suicide management in a rural district of Kwa-Zulu-Natal. *South Afr J Psychiatry*, 13: 52-5. |
| **Nordentoft 2005** | Denmark | Cross-sectional | 223 | Persons (61.4% female), age range not reported (mean: 37.8 years), presenting to the emergency department of any one of four general hospitals following a suicide attempt. | NR | NR | NR | Bispebjerg Hospital, Rigshospitalet, Frederiksberg Hospital and Hvidovre Hospital, Copenhagen, Denmark. | 01/02/2001-01/05/2001 | Psychiatric evaluation in ED or ICU or psychiatric emergency unit by the duty psychiatrist. | Nordentoft M, et al. (2005). Registrat-ion, psychiatric evaluation and adherence to psychiatric treatment after suicide attempt. *Nord J Psychiatry*, 59: 213-6. |
| **Olfson 2012**  ***(Bridge 2012)*^[[39]](#footnote-39)^**  ***(Olfson 2013)*^[[40]](#footnote-40)^** | USA | Cohort | 4,866  *(7,355)* | Persons (68.2% female), aged 21 to 64 years (average: not reported), presenting to any emergency department in the country following an episode of DSH. | 32.8 | NR | 51.1 | Medicaid 2006 database, USA | 03/03/2006-01/12/2006 | Mental health assessment in ED. Unclear who undertook the assessment. | Olfson M, et al. (2012). Emergency treatment of deliberate self-harm. *Arch Gen Psychiatry*, 69: 80-8. |
| **Opmeer 2017** | UK | Cohort | 318  *(381)* | Persons (63.2% female), age range not reported (mean: 34.5±14.5 years), presenting to the emergency department of "a large hospital…" (p.1) following DSH. | NR | NR^*^ | NR | Bristol Self-harm Monitoring System, Bristol, UK | 2014 cohort:  01/01/2014-31/03/2014  2015 cohort:  01/01/2015-31/03/2015 | Psychosocial assessment in ED by the duty psychiatrist and/or mental health trained staff. | Opmeer BC, et al. (2017). Extending the liaison psychiatry service in a large hospital in the UK: A before and after evaluation of the economic impact and patient care following ED attendances for self-harm. *BMJ Open*, 7: e016906. |
| **Owens 1988** | UK | Cohort | 496  *(525)* | Persons (63.4% female), aged 14 years and older (average: not reported), presenting to the emergency department of a general medical hospital following an episode of DSP. | NR | 14.1 | 44.2 | Nottingham Hospital, Nottingham, UK | 01/04/1982-31/07/1982 | Psychiatric consultation in ED by the duty psychiatrist. | Owens DW, et al. (1988). The accident and emergency department management of deliberate self-poisoning. *Br J Psychiatry*, 152: 830-3. |
| **Pang 1996** | UK | Cross-sectional | 155 | Persons (percent female not reported), aged 38 to 85 years (mean: 33.7±13.2 years), referred to the emergency department following an episode of DSH. | NR | NR | NR | One unnamed teaching hospital in central London, UK. | 01/06/1991-30/11/191 | Psychosocial assessment in ED by social workers or psychiatrists in specialist team, or by duty psychiatrist. | Pang AHT, et al. (1996). Audit of a multi-disciplinary assessment unit for deliberate self-harm patients in a general hospital. *Arch Suicide Res*, 2: 207-12. |
| **Pavarin 2014** | Italy | Cohort | 505  *(788)* | Persons (60.6% female), aged 13 years and older (mean: 45.6 years; SD: not reported), admitted to the emergency department of any general hospital following a suicide attempt. | NR | NR | 43.3 | Any of the general hospitals in metro-politan area of (with the exception of one: Sant'Orsola Hospital), Bologna, Italy. | 01/01/2004-31/12/2010 | Pyschiatric assessment/coun-sulation in ED by the duty psychiatrist. | Pavarin RM, et al. (2014). Emergency department admission and mortality rate for suicidal behavior. *Crisis*, 35: 406-14. |
| **Perquier 2017** | France | Cross-sectional | 168  *(355)* | Persons (66.1% female), aged 24 to 53 years (mean: 38.7±14.3 years), presenting to the emergency department of a general academic hospital and subsequently referred to the psychiatric emergency department (as per local protocols) following an episode of suicide attempt. | NR | 50.6 | NR | Bichat-Claude Bernard University Hospital Centre, Paris, France. | 01/03/2015-30/05/2016 | Psychiatric assessment by the duty psychiatrist and/or mental health trained staff. | Perquier F, et al. Suicide attempters examined in a Parisian Emergency Department: Contrasting characteristics associated with multiple suicide attempts or with the motive to die. *Psychiatr Res*, 253: 142-9. |

| **Reith 2004**  ***(Carter 2005a)*^[[41]](#footnote-41)^**  ***(Carter 2005b)*^[[42]](#footnote-42)^**  ***(Carter 2006)*^[[43]](#footnote-43)^**  ***(Hiles 2015)*^14^**  ***(Ticehurst 2002)*^[[44]](#footnote-44)^**  ***(Whyte 1997)*^[[45]](#footnote-45)^** | Australia | Cohort | 4,105  *(584)* | Persons (58.9% female), age range not reported (average: not reported), presenting to the emergency department of a general medical hospital and admitted to a toxicology service per local protocols following an episode of DSP. Note, data from Ticehurst (2002) included only for analyses related to persons aged 65 years and older. Note, data from Whyte (1997) included in analyses related to events. | NR | NR | NR | Hunter Area Toxicology Service, Newcastle, Australia | 01/01/1991-31/12/2000 | Psychiatric assessment in toxicology unit. Unclear who undertook the assessment. | Reith DM, et al. (2004). Risk factors for suicide and other deaths following hos-pital treated self-poisoning in Australia. *Aust N Z J Psychiatry*, 38: 520-5. |
| --- | --- | --- | --- | --- | --- | --- | --- | --- | --- | --- | --- |
| **Runeson 2000** | Multicounty: Italy and Sweden | Cross-sectional | 119 | Persons (62.4% female), aged between 16 and either 89 years (Sweden) or 94 years (Italy) (mean: 38.0 years; SD: not reported), presenting to one of two general hospitals following an episode of self-harm. | NR | 47.7 | 34.5 | Padua Hospital, Padua, Italy and Huddinge Hospital, Stockholm, Sweden. | 01/09/1991-30/06/1996 | Psychiatric assessment in ED by the duty psychiatrist and/or mental health trained staff. | Runeson B, et al. (2000). Management of suicide attempts in Italy and Sweden: A comparison of services offered to consecutive samples of suicide attempters. *Gen Hosp Psychiatry*, 22: 432-6. |
| **Runeson 2001** | Sweden | Cross-sectional | 309  *(329)* | Persons (50.8% female), aged 10 to 89 years (mean: 38.1 years; SD: not reported), presenting to a general hospital following an episode of DSH. | NR | 28.9 | 59.0 | Skaraborg County Hospital (campus not named), Sweden. | 15/08/1994-14/08/1995 | Specialist psychosocial assessment by the duty psychiatrist. Setting unclear. | Runeson B. (2001). Parasuicides without follow-up. *Nord J Psychiatry*, 55: 319-23. |
| **Russell 2010** | UK | Cohort | 675  *(787)* | Persons (56.4% female), aged 16 to 99 years (median: 32.0 years), presenting to the emergency department of a single acute hospital following an episode of DSH. | NR | NR | 47.4 | Bradford Royal Infirmary, West Yorkshire, UK | 01/01/2002-31/07/2003 | Specialist psychosocial assessment in ED by the duty psychiatrist and/or mental health trained staff. | Russell G, et al. (2010). Psychosocial assessment following self-harm: Repetition of nonfatal self-harm after assessment by psychiatrists or mental health nurses. *Crisis*, 31: 211-6. |
| **Rygnestad 1991** | Norway | Cohort | 618 | Persons (57.6% female), aged 13 years and older (mean: 35.3 years; SD: not reported), admitted to a general regional hospital following an episode of DSP. Note, data disaggregated by gender/sex group. | NR | 26.4 | 78.5 | Regional Hospital of Trondheim, Norway. | 1978 cohort: 01/01/1978-31/12/1978  1987 cohort: 01/01/1987-31/12/1987 | Psychiatric evaluation by the duty psychiatrist. | Rygnestad T, et al. (1991). Epidemi-ological, social and psychiatric aspects in self-poisoned patients. *Soc Psych-iatry Psychiatr Epidemiol*, 26: 53-62. |
| **Scott 1993** | UK | Case-control | 16 | Persons (43.8% female), aged 12 to 20 years (mean: 17.7 years; SD: not reported), presenting to the emergency department of a general medical hospital following DSH. | NR | 43.8 | NR | Dumfries and Galloway Royal Infirmary, Scotland, UK. | 01/01/1988-31/12/1988 | Psychiatric assessment in ED. Unclear who undertook the assessment. | Scott SW, et al. (1993). Adolescent self-mutilation in a rural area. *J Adolesc*, 16: 101-5. |
| **Shahid 2009** | Pakistan | Cross-sectional | 98 | Persons (63.3% female), age range not reported (mean: 23.5 years; SD: not reported), presenting to the emergency department of a University Hospital following an episode of DSH. | NR | 7.1 | 24.2 | Aga Khan University Hospital, Karachi, Pakistan. | 01/01/2004-31/12/2004 | Psychiatric evaluation in ED by the duty psychiatrist. | Shahid M, et al. (2009). Deliberate self-harm in the emergency department: Experience from Karachi, Pakistan. *Crisis*, 30: 85. |
| **Shekunov 2021** | USA | Case series | 97 | Children and adolescents (17.5% female), aged 0 to 18 years (mean: 15.6±2.1 years), evaluated for excessive acetaminophen/ paracetamol exposure. Authors further disaggregated data by intentionality, enabling inclusion of data for those engaging in intentional acetaminophen/ paracetamol overdose only in this review. | NR | 22.7 | NR | Olmstead County Hospital, Roch-ester, Minnesota, USA. | 01/01/2004-31/12/2010 | Psychiatric consultation. Unclear who undertook the assessment and setting. | Shekunov J, et al. (2021). Clinical characteristics, outcomes, disposition, and acute care of children and adoles-cents treated for acetaminophen toxicity. *Psychiatr Serv*, 72: 758-65. |
| **Shuchman 1996** | USA | Cohort | 28 | Children and adolescents (57.1% female), aged 12 to 18 years (mean: 15.0 years; SD: not reported), hospitalised at a level 1 trauma centre following a suicide attempt. | NR | NR | NR | San Francisco General Hospital, San Fran-cisco, California, USA. | 01/01/1991-31/12/1991 | Psychosocial assessment. Unclear who undertook the assessment. | Shuchman M, et al. (1996). Intervent-ions among adolescents who were violently injured and those who attempted suicide. *Psychiatr Serv*, 47: 755-7. |
| **Song 2012** | South Korea | Cohort | 335 | Persons (percent female not reported), aged 18 years and older (average: not reported), presenting to one of two academic hospital emergency departments following a suicide attempt. | NR | NR | NR | Boramae Medical Center, Seoul, South Korea. | 01/06/2011-30/04/2012 | Pyschiatric evaluation/consultation in ED by the duty psychiatrist. | Song KJ, et al. (2012). Implementation of ED based in-depth surveillance for suicidal attempt: Descriptive study. *J Emerg Med*, 43: 923. |
| **Suominen 2000** | Finland | Case-control | 111 | Persons (55.0% female), aged 15 years and older (mean: 37.3±12.6 years), referred to the emergency department of any one of four general hospitals following a suicide attempt. | NR | 65.8 | 68.5 | All hospitals, Helsinki, Finland. | 01/01/1990-31/07/1990 | Psychiatric assessment. Unclear who undertook the assessment. | Suominen KH, et al. (2000). Suicide attempts and personality disorder. *Acta Psychiatr Scand*, 102: 118-25. |
| **Suominen 2004a**  ***(Suominen 2002)*^[[46]](#footnote-46)^**  ***(Suominen 2004b)*^[[47]](#footnote-47)^**  ***(Suominen 2004c)*^[[48]](#footnote-48)^** | Finland | Cohort | 1,198 | Persons (52.7% female), aged 15 years and older (mean: 38.1±13.3 years), presenting to the emergency department of any one of four general hospitals follow-ing a suicide attempt. Note, data from Suominen (2004c) included for analyses related to older persons only. | NR | 57.3 | NR | All hospitals, Helsinki, Finland. | 15/01/1997-14/01/1998 | Psychiatric consultation in ED. Unclear who undertook the assessment. | Suominen K, et al. (2004). Substance use and male gender as risk factors for deaths and suicide: A 5-year follow-up study after deliberate self-harm. *Soc Psychiatry Psychiatr Epidemiol*, 39: 720-4. |

| **Sztajnkrycer 2007** | USA | Cross-sectional | 148 | Persons (percent female not reported), aged 15 years and older (average: not reported), presenting to the emergency department of a tertiary care hospital following an episode of intentional drug overdose. | NR | NR | NR | St Mary's Hospital, Rochester, Minnesota, USA. | 01/10/2002-30/11/2002 | Psychiatric evaluation in ED. Unclear who undertook the assessment. | Sztajnkrycer MD, et al. (2007). Development and implementation of an emergency department observation unit protocol for deliberate drug ingestion in adults – preliminary results. *Clin Toxicol*, 45: 499-504. |
| --- | --- | --- | --- | --- | --- | --- | --- | --- | --- | --- | --- |
| **Tountas 2001** | Greece | Cross-sectional | 146 | Persons (65.8% female), aged 15 to 74 years (mean: 30.9±11.9 years), admitted to a Regional General Hospital following an episode of self-poisoning. | NR | 45.2 | 31.5 | Regional General Hospital of Nikea, Greece. | 01/11/1999-30/11/2000 | Mental status examination by duty psychiatrist. Unclear who undertook the assessment. | Tountas C, et al. (2001). Voluntary self-poisoning as a cause of admission to a tertiary hospital internal medicine clinic in Piraeus, Greece within a year. *BMC Psychiatry*, 1: 4. |
| **Vidalis 1987** | UK | Cross-sectional | 93 | Persons (64.5% female), aged 10 to 84 years (average: not reported), presenting to the emergency department receiv-ing an admissions diagnosis of intentional overdose. | NR | NR | NR | NR | NR | Psychiatric assessmentin ED by the duty psychiatrist. | Vidalis AA, et al. (1987). Self-poison-ing: Could psychiatric management by improved? *Int J Soc Psychiatry*, 33: 312-5. |
| **Whyte 2001** | UK | Cross-sectional | 50 | Persons (percent female not reported), aged 16 years and older (average: not reported), presenting to A&E following an episode of DSH. | NR | NR | NR | Kettering General Hospital, Northamptonshire, UK | 01/03/1999-31/12/1999 | Psychosocial assessment in ED by the duty psychiatrist and/or mental health trained staff. | Whyte S, et al. (2001). Deliberate self-harm: The impact of a specialist DSH team on assessment quality. *Psychiatr Bull*, 25: 98-101. |
| **Witt 2023** | Australia | Cross-sectional | 5,428  *(7,736)* | Persons (54.8% female), aged 9 years and older (median: 30.0 years, IQR: 23.0 to 44.0 years), presenting to the ED following an episode of DSH. | NR | NR | NR | Royal Melbourne Hospital, Melb-ourne, Australia. | 01/01/2012-31/12/2019 | Psychiatric consultation in ED by emergency mental health trained staff. | Witt K, et al. (2023). Characteristics of self-harm presentations to the emergency department of the Royal Melbourne Hospital, 2012-19: Data from the Self-Harm Monitoring System for Victoria. *Aust Emerg Care*, ePub ahead of print, DOI: 10.1016/j.auec.2023.01.003. |

**Notes:** Secondary samples denoted by italicised text. A&E: accident and emergency department; ED: emergency department; ER: emergency room; DSH: deliberate self-harm; DSP: deliberate self-poisoning; Hx: history; IQR: inter-quartile range; *N*: number of individuals; ICD-10; International Classification of Disease, 10^th^ edition; *N*(events): number of events; NHS: National Health Service, UK; NR: not reported; RCT: randomised controlled trial; SES: socio-economic status; TAU: treatment as usual; UAE: United Arab Emirates; UK: United Kingdom; USA: United States of America.

**Figure SD2.** Risk of bias summary ratings for all included studies.

| 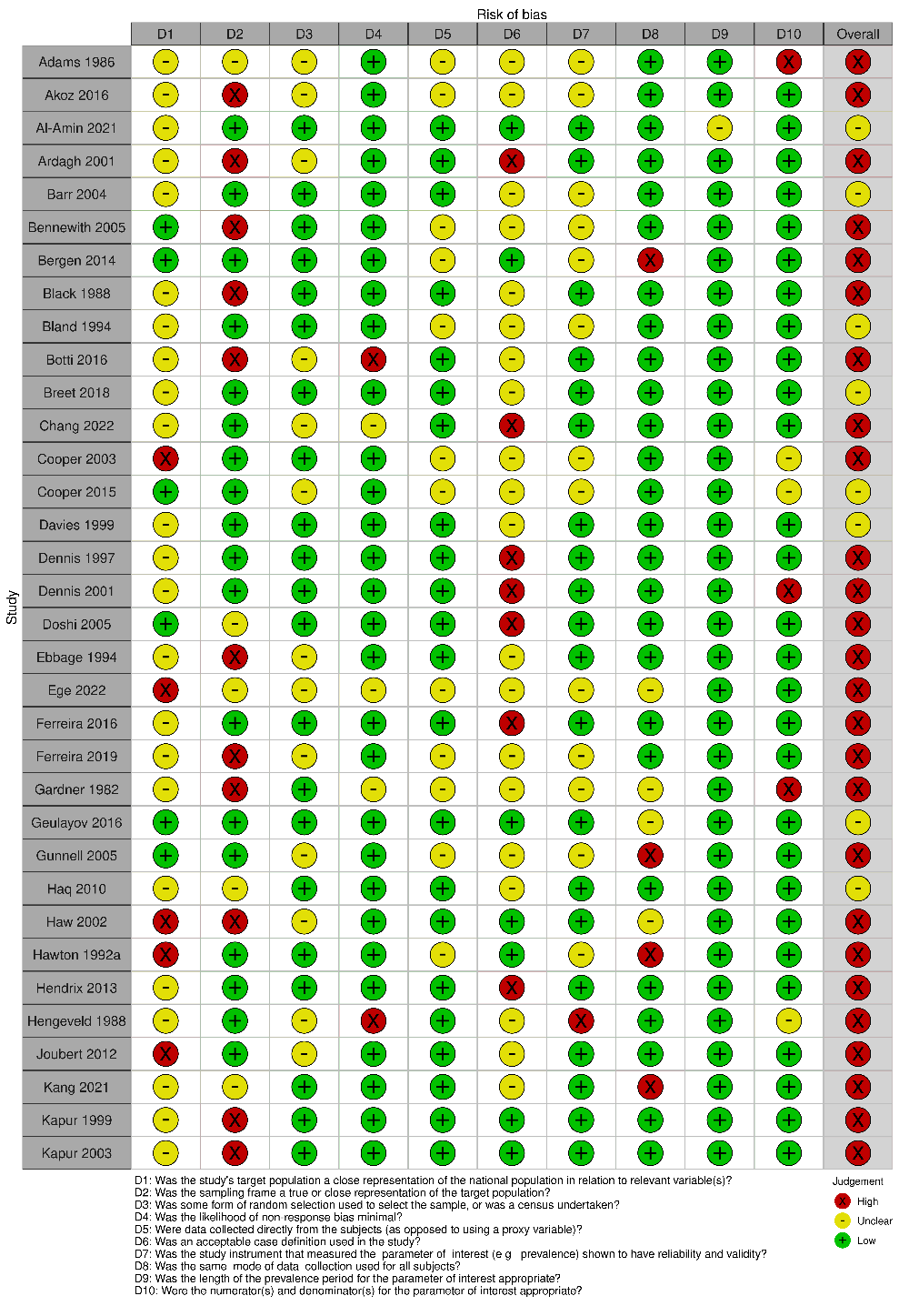 | 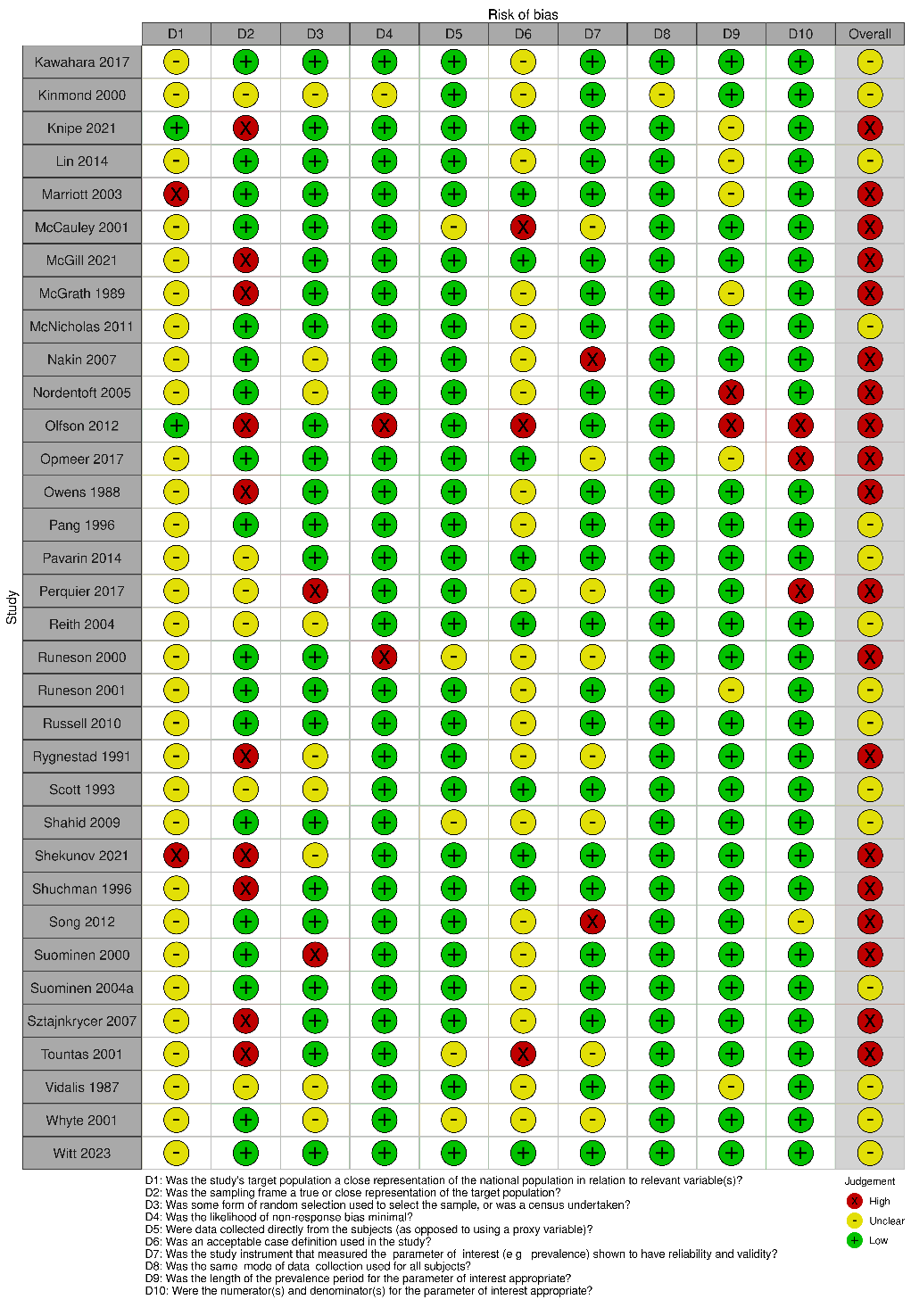 |
| --- | --- |

**Table SD3.** Risk of bias supporting statements for all included studies.

| **Study** | **1**  **Was the study’s target population a close representation of the national population in relation to relevant variables(s)?** | **2**  **Was the sampling frame a true or close representation of the target population?** | **3**  **Was some form of random selection used to select the sample, or, was a census undertaken?** | **4**  **Was the likelihood of non-response bias^1^ minimal?** | **5**  **Were data collected directly from the subjects (as opposed to using a proxy variable)?** | **6**  **Was an acceptable case definition used  in the study?** | **7**  **Was the study instrument that measured the prevalence shown to have reliability and validity?** | **8**  **Was the same mode of data collection used for all subjects?** | **9**  **Was the length of the prevalence period appropriate?** | **10**  **Were the numerator(s) and denominator(s) for the prevalence appropriate?** |
| --- | --- | --- | --- | --- | --- | --- | --- | --- | --- | --- |
| **Adams 1986** | The study was conduct-ed in "[t]he Norfolk and Norwich Hospital serves a population of 450 000 (1982), catering as it does for the needs of Norwich City (136 000) and the greater part of the county of Norfolk, excluding the catchment areas of King’s Lynn and Greater Yarmouth" (p.5). However, it is unclear whether this is representative of the national population. | All participants aged 12 years and older who engaged in DSP were eligible for inclusion. Only those "whose notes could not be traced" (p.5) were excluded. However, those engaging in other methods of DSH would also have been excluded. | NR | "Excluded from the study...[were] those (40) whose notes could not be traced" (p.5), giving an effective non-response rate of 0.5%. | NR | NR | NR | No specific inform-ation reported, how-ever, it is likely that the same mode of data collection was used for all partici-pants. | The study reported outcome(s) at pre-sentation and/or immediately post-discharge and so it is likely the length of the prevalence period was appro-priate. | Denominators for each outcome of interest could only be calculated from percentages, leading to some inconsistencies in the denominator between outcomes and a failure to match grand denominator in some instances. |
| **Akoz 2016** | This study was conduc-ted in a "...university hospital located in Erzurum in the Eastern region of Turkey" (p.832), however, it is unclear whether this is representative of the national population. | "Patients who engaged in mutilation without suicidal intent and repetitive superficial bodily harm without suicidal intent were excluded" (p.832). | NR | No specific infor-mation reported, however, data for all eligible participants were included in analyses. | Whilst "[i]nformation was collected pro-spectively on a specially designed data collection form" (p.832), it is unclear whether this form was completed using data from self-report and/or hospital/ medical records for the outcomes of relevance to this review. | Whilst, in this study, "[a] suicide attempt is defined as an act with a non-fatal out-come in which an individual deliberat-ely ingests a substan-ce in excess of the prescribed or gen-erally recognized therapeutic dosage" (p.832), it is unclear whether this deter-mination was made on the basis of diagnostic codes alone, or whether additional review was undertaken to investigate intent in ambiguous cases. | NR | No specific inform-ation reported, how-ever, it is likely that the same mode of data collection was used for all partici-pants. | The study reported outcome(s) at pre-sentation and/or immediately post-discharge and so it is likely the length of the prevalence period was appro-priate. | There were no errors in the reporting of the numerator(s) and denominator(s) for the prevalence. |

| **Al-Amin 2021** | "HMC is the leading state health care center in Qatar...the ED is the only state emergency facility in Doha" (p.316). However, it is unclear whether this is represent-tative of the national population. | "...at the time of the study, private sector emergency services, of which there were very few, would refer all self-harm cases to HMC ED. Therefore, we believe that the sample assessed was largely representative of the population under study" (p.317). | "This is a retrospective study of all the cases presented [sic] to the Emergency Depart-ment…" (p.316). | No specific infor-mation reported, however, data for all eligible participants were included in analyses. | Data were collected "...using the medical records available" (p.317). | "Self-harm and suic-idal attempts were identified by em-ploying a thorough, systematic search method, which con-sisted of filtering cases based on the ICD 10 codes…and utilizing relevant search words (such as suicide, overdose, ingestion, jumping" (p.317). Combining ICD-10 diagnostic codes with additional data fields has been shown to improve the accuracy of identification of DSH.^[[49]](#footnote-49)^ | Hospital/medical records used. | No specific inform-ation reported, how-ever, it is likely that the same mode of data collection was used for all partici-pants. | NR | There were no errors in the reporting of the numerator(s) and denominator(s) for the prevalence. |
| --- | --- | --- | --- | --- | --- | --- | --- | --- | --- | --- |
| **Ardagh 2001** | Whilst "Christchurch Hospital provides a sole Emergency Department servicing approximately 330 000 patients in the Christchurch urban area..." (p.423), it is unclear whether this is representative of the national population. | "...all patients with a discharge diagnosis of DSP" were included. Therefore, those engaging in other methods would have been excluded. | NR | No specific infor-mation reported, however, data for all eligible participants were included in analyses. | "The Emergency Department records and inpatient hospital notes were reviewed..." (p.423). | "…the computer database was used to identify [cases] with a discharge diagnosis of DSP" (p.423). Ascertainment of DSH from diagnostic or discharge codes alone has been shown to under-enumerate cases.^49^ | Hospital/medical records used. | No specific inform-ation reported, how-ever, it is likely the same mode of data collection was used for all participants. | The study reported outcome(s) at pre-sentation and/or immediately post-discharge and so it is likely the length of the prevalence period was appro-priate. | There were no errors in the reporting of the numerator(s) and denominator(s) for the prevalence. |
| **Atreya 2015** | "The research was con-ducted in a tertiary care teaching hospital of Nepal" (p.35). However, no specific information is reported from which hospitals participants were recruited. There-fore, it is difficult to determine whether participants were recruited from a region representative of the national population. | Only "...hanging cases who presented to the ED" (p.36) were included. Therefore, those engaging in other methods would have been excluded. | NR | No specific infor-mation reported, however, data for all eligible participants were included in analyses. | Information was co-llected following "...detailed exam-ination of treatment files…" (p.36). | "The cases were id-entified based on a manual search of the patient's registration log book. The hospital number of the cases marked as near hanging cases was noted and relevant files were obtained..." (p.36). | Hospital/medical records used. | No specific inform-ation reported, how-ever, it is likely the same mode of data collection was used for all participants. | The study reported outcome(s) at pres-entation and/or immediately post-discharge and so it is likely the length of the prevalence period was appro-priate. | There were no errors in the reporting of the numerator(s) and denominator(s) for the prevalence. |

| **Barr 2004** | "Data were collected... from a District General Hospital that served a mixed rural/urban population of approx-imately 250,000 in North Wales" (p.109). However, it is not clear whether this region is representative of the national population. | "Because the unit under study is relative-ly geographically isolated from other general hospitals it is probable that almost all patients referred for A&E care from the surrounding area would have attended this particular hospital and so will appear in the data reported here" (p.110). | "The study is part of a[n]…analysis of all DSH presentations…" (p.109). | No specific infor-mation reported, however, data for all eligible participants were included in analyses. | "Where possible, data were collected in face-to-face inter-views with patients. When this was not possible, such as those cases where patients had already been discharged, were unconscious or had been judged not to require specialist psychosocial assess-ment, A&E casualty cards and hospital case notes were searched" (p.109). | Whilst "[t]he diag-nosis of deliberate self-harm included any act of non-accidental self-harm, such as drug over-dose, consumption of a noxious sub-stance, self-laceration, hang-ing or gassing" (p.109), it is unclear whether this deter-mination was made on the basis of diagnostic codes alone, or whether additional review was undertaken to investigate intent in ambiguous cases. | NR | No specific inform-ation reported, how-ever, it is likely the same mode of data collection was used for all participants. | The study reported outcome(s) at pres-entation and/or immediately post-discharge and so it is likely the length of the prevalence period was appro-priate. | There were no errors in the reporting of the numerator(s) and denominator(s) for the prevalence. |
| --- | --- | --- | --- | --- | --- | --- | --- | --- | --- | --- |
| **Bennewith 2005** | "Data were collected on all self-harm attendances at 32 hospitals in Eng-land...The aim of the selection process...was to ensure that the hos-pitals studied were a nationally representative sample of all hospitals in England providing an Accident and Emer-gency service" (p.92). | "Data from 10 hos-pitals were excluded from this analysis as information on the reason for a psycho-social assessment not being carried out was missing for more than 10% of episodes (9 hospitals) or was only available in aggregated form (one hospital). The two largest hos-pitals in the study were among the 10 ex-cluded." (p.92). Additionally, there is evidence that the proportion of episodes resulting in psycho-social assessment was lower in excluded hospitals (50.7%) as compared with included hospitals (59.0%). | "Data were collected on all self-harm attendances…" (p.92). | No specific infor-mation reported, however, data was missing for only 0.6% of cases. | Whilst data "…were collected using a one-page form and, where a psychosocial ass-essment had not been carried out, infor-mation on the reasons for non-assessment was requested" (p.92), it is unclear whether this infor-mation was ascertain-ed from participant self-report and/or hospital/medical records for the outcomes of rele-vance to this review. | NR | NR | No specific inform-ation reported, how-ever, it is likely the same mode of data collection was used for all participants. | The study reported outcome(s) at pres-entation and/or immediately post-discharge and so it is likely the length of the prevalence period was appro-priate. | There were no errors in the reporting of the numerator(s) and denominator(s) for the prevalence. |
| **Bergen 2014** | "The study was under-taken in three centres in the Multicentre Study of SH in England..." (p.2). These centres have pre-viously been shown to have good representation of the national population. | "Data were collected on all individuals who presented with non-fatal SH…" (p.2). Only "...1285(3.2%) aged under 15 years and 47 (0.1%) with unknown age were excluded from analyses" (p.3). | "...Data were collected on all individuals who presented with non-fatal SH..." (p.2). | "Individuals (940, 2.3%) not traced were excluded..." (p.3). | Whilst "...data on each episode (inc-luding referral for aftercare) were col-lected by clinicians" (p.2), it is unclear whether this infor-mation was ascertain-ed from participant self-report and/or hospital/medical records for the out-comes of relevance to this review. | "Non-fatal SH was defined as intentional self-poisoning or self-injury, irrespect-ive of motivation or degree of suicidal intent" (p.2). Pre-vious work using data from the Multi-centre Study of Self-harm in England has clarified that cases are ascertained following review of hospital/medical records. | NR | Given that "[p]atients not receiving an ass-essment were ident-ified through scrutiny of ED and medical records, from which more limited data were extracted by re-search clerks, or in one centre by clin-icians…" (p.2), it would appear that different modes of data collection were used. | The study reported outcome(s) at pres-entation and/or immediately post-discharge and so it is likely the length of the prevalence period was appropriate. | There were no errors in the reporting of the numerator(s) and denominator(s) for the prevalence. |
| **Black 1988** | "The notes of patients presenting to the hospital following an overdose were examined..." (p.97), however, no specific information is reported from which hospitals participants were recruited. Therefore, it is difficult to determine whether participants were recruited from a region representative of the national population. | "...self-poisoning pat-ients seen in the cas-ualty department" (p.97). However, those engaging in other methods would have been excluded. | "Case notes and cas-ualty cards were stud-ied for all self-poison-ing patients seen in the casualty department" (p.97). | No specific infor-mation reported, however, data for all eligible participants were included in analyses. | "Case notes and cas-ualty cards were studied..." (p.97). Furthermore, "[a]tt-empts to gain further information (from relatives, social workers, or hostel staff who know the patient) were record-ed in the notes of 3% of the casualty officers" (p.98). | NR | Hospital/medical records used. | No specific inform-ation reported, how-ever, it is likely the same mode of data collection was used for all participants. | The study reported outcome(s) at pres-entation and/or immediately post-discharge and so it is likely the length of the prevalence period was appro-priate. | There were no errors in the reporting of the numerator(s) and denominator(s) for the prevalence. |
| **Bland 1994** | "A separate study of all the Edmonton hospitals established that 20% of all parasuicides were treated at the University of Alberta Hospitals" (p.392). However, it is unclear whether this region is representative of the national population. | "...all records of para-suicides attending the University of Alberta Hospitals..." (p.391) were included. There were no specific exclusion criteria. | "...all persons...who attended the emerg-ency department...for parasuicide were examined" (p.392). | "Some data were missing on one case, and therefore some analyses use 274 cases" (p.392), giv-ing an effective non-response rate of no more than 0.4%. | NR | NR | NR | No specific inform-ation reported, how-ever, it is likely the same mode of data collection was used for all participants. | The study reported outcome(s) at pre-sentation and/or immediately post-discharge and so it is likely the length of the prevalence period was appro-priate. | There were no errors in the reporting of the numerator(s) and denominator(s) for the prevalence. |
| **Botti 2016** | Whilst this study was conducted in "...the Toxicological Unit of Florence Careggi Uni-versity Hospital..." (p.S461) in Florence, Italy, it is unclear whether this region is representative of the national population. | Only those engaging in "...DSH by poisoning diagnosis...were stud-ied" (p.S461). There-fore, those engaging in other methods would have been excluded. | NR | Whilst "[t]here were 252 patients in which psychiatric evaluation was performed in 157 (62.3%)" (p.S461), outcomes were only reported for the 62.3% of the sample with complete ass-essment information, giving an effective non-response rate of 37.7%. | "We analyzed clinical records, including psychiatric evalu-ation when available" (p.S461). | NR | Hospital/medical records used. | No specific inform-ation reported, how-ever, it is likely the same mode of data collection was used for all participants. | The study reported outcome(s) at pre-sentation and/or immediately post-discharge and so it is likely the length of the prevalence period was appro-priate. | There were no errors in the reporting of the numerator(s) and denominator(s) for the prevalence. |

| **Breet 2018** | "The aim of this study was to collect epidem-iological data...in an urban hospital in the Western Cape Province of S[outh] A[frica]..." (p.2). However, it is unclear whether this region would be representative of the national population. | "...consecutive pre-sentations of self-harm ...were eligible for inclusion. Cases were excluded if: the files were missing or there was not sufficient in-formation available in the patient file (17 patients); the patient had already been inc-luded in the sample on a prior presentation to the hospital during the period of data col-lection (9 patients); patients left the hos-pital before data was captured (1 patient); or patients died as a result of their injuries (5 patients)" (p.2). | "Data were collected for this…study from… consecutive self-harm patients presenting to the ED of a hospital" (p.2). | "...[c]ases were ex-cluded if: the files were missing or there was not suff-icient information available in the patient file (17 patients)…" (p.2), giving an effective non-response bias of 6.7%. | "The data was col-lected from patient records that contain-ed information recorded by doctors in the ED…" (p.3). | Whilst "[i]n this study self-harm is defined as intentional self-injury or self-poisoning with non-fatal outcome, re-gardless of the de-gree of intent to die, which is deliberate and is non-habitual" (p.2), it is unclear whether this deter-mination was made on the basis of diag-nostic codes alone, or whether additional review was under-taken to investigate intent in ambiguous cases. | Hospital/medical records used. | No specific inform-ation reported, how-ever, it is likely the same mode of data collection is used for all participants. | The study reported outcome(s) at pre-sentation and/or immediately post-discharge and so it is likely the length of the prevalence period was appro-priate. | There were no errors in the reporting of the numerator(s) and denominator(s) for the prevalence. |
| --- | --- | --- | --- | --- | --- | --- | --- | --- | --- | --- |
| **Chang 2022** | “Taipei Tzu Chi Hos-pital, a regional hospital in urban northern Taiwan, and Chi Mei Medical Center, a tertiary center in suburban southern Taiwan” (p.132). However, it is unclear whether this region would be representative of the national population. | “Children under 18 years who had one or more eligible ICD-10 codes and a document-ted self-inflicted injury on emergency depart-ment presentation were included” (p.132). Only “[v]isits without a documented self-inflicted injury at the emergency department were excluded” (p.132). | NR | NR | “The search was performed using ASUS Intelligent Cloud Service (AICS), a search engine and database recording anonym-ized demographic and clinical data” (p.132). | Whilst “[s]elf-inflic-ted injury referred to all injuries that were intentionally self-directed, including intentional poison-ing, cut, pierce, suffocation, fall, battery, firearm, and burn, regardless of suicidal ideation” (p.132), episodes were identified from “International Classi-fication of Diseases Tenth Revision (ICD-10) codes of suicide attempt (T14.91), intentional self-harm (X71-83), sequelae of intent-ional self-harm (Y87.0), and intent-ional poisoning (T36-71)” (p.132). Ascertain-ment of DSH from diagnostic or dis-charge codes alone has been shown to under-enumerate cases.^49^ | Hospital/medical records used. | No specific inform-ation reported, how-ever, it is likely the same mode of data collection is used for all participants. | The study reported outcome(s) at pre-sentation and/or immediately post-discharge and so it is likely the length of the prevalence period was appro-priate. | There were no errors in the reporting of the numerator(s) and denominator(s) for the prevalence. |

| **Cooper 2003** | "The Manchester and Salford Self-harm (MASSH) project... covers a mainly urban population of approx.-imately 500 000 people, with large areas of socio-economic deprivation" (p.284). It is therefore unlikely that this region is representative of the national population. | "The MASSH project collects data on DSH by individuals aged 10 years or above... Episodes are [only] not included if the patient did not wait for assess-ment or refused treat-ment, because forms cannot be completed in this case" (p.284). | No specific inform-ation reported, how-ever, "[a] recent audit of ED case records showed that the project correctly identified over 95% of DSH presentations" (p.285). | "...assessors failed to complete...the form" (p.285) in 9.3% of cases. | Whilst "[o]n each episode of self-harm the assessing doctor completes a standard assessment form. The form provides a structure for the ass-essment of… initial clinical management and follow-up arrang-ements" (p.284), it is unclear whether this information was as-certained from parti-cipant self-report and/or hospital/ medical records for the outcomes of relevance to this review. | Whilst "DSH is defined in accord-ance with the World Health Organization/ Euro Multi-Centre Study on Para-suicide" (p.284), it is unclear whether this determination was made on the basis of diagnostic codes alone, or whether additional review was undertaken to investigate intent in ambiguous cases. | NR | No specific inform-ation reported, how-ever, it is likely the same mode of data collection was used for all participants. | The study reported outcome(s) at pre-sentation and/or immediately post-discharge and so it is likely the length of the prevalence period was appro-priate. | Data for one outcome may be incomplete as the numerator for those who received treatment was not disaggregated into those receiving medical treatment and those receiving psychiatric treatment. |
| --- | --- | --- | --- | --- | --- | --- | --- | --- | --- | --- |
| **Cooper 2015** | "A random sample of 32 hospitals in England... participated in the current study" (p.102). Therefore, this study is representative of the national population. | "...individuals aged 18 years or over [presen-ting] with...episodes of self-harm" (p.103) were included. There were no specific exclusion criteria. | Whilst "[a] random sample of 32 hospitals in England...partici-pated" (p.102), no specific information was reported on the method(s) used to select participants. | "Due to limited acc-ess to data at one of the sites, 31 [of 32] hospitals were inc-luded in the analysis for this paper" (p.102), giving an effective non-response rate of 3.1%. | Whilst "[c]linical management of self-harm episodes was categorised into the following: specialist mental health assess-ment, admission to a general medical bed, psychiatric admiss-ion, specialist comm-unity mental health follow-up and ref-erral to non-statutory services" (p.102), it is unclear whether this information was as-certained from parti-cipant self-report and/or hospital/med-ical records for the outcomes of rele-vance to this review. | Whilst "[s]elf-harm was defined as a deliberate non-fatal act of self-injury or self-poisoning, done in the knowledge that it was potenti-ally harmful and in the case of drug overdose that the amount taken was excessive..." (p.102), it is unclear whether this determination was made on the basis of diagnostic codes alone, or whether additional review was under-taken to investigate intent in ambiguous cases. | NR | No specific inform-ation reported, how-ever, it is likely the same mode of data collection was used for all participants. | The study reported outcome(s) at pre-sentation and/or immediately post-discharge and so it is likely the length of the prevalence period was appro-priate. | Denominators differed across outcomes and no explanation was provided, suggesting variable non-response across hospital sites. |
| **Davies 1999** | "Altnagelvin Area Hos-pital, Derry provides the only accident and emer-gency department in the Foyle Trust area which serves a population of approximately 150,000 people in the north-west of Northern Ireland" (p.10). However, it is unclear whether this region is representative of the national population. | "...admissions (aged 16 years and younger) ...as a result of para-suicide" (p.10) were included. There were no explicit exclusion criteria. | The study included "all admissions..." (p.10). | No specific inform-ation reported, how-ever, data for all eligible participants were included in analyses. | "The information pre-sented in this paper was gathered from an array of written records. At no time were any individuals interviewed." (p.10). | NR | Hospital/medical records used. | No specific inform-ation reported, how-ever, it is likely the same mode of data collection was used for all participants. | The study reported outcome(s) at pre-sentation and/or immediately post-discharge and so it is likely the length of the prevalence period was appro-priate. | There were no errors in the reporting of the numerator(s) and denominator(s) for the prevalence. |

| **Dennis 1997** | "...attendances at the Leicester Royal Infirm-ary A&E department..." (p.311) were included. However, it is not clear if this is representative of the national population. | "…records for attend-ances…with a diag-nosis of 'self-inflicted' injury" (p.311) were included. Only those "658 cases...[that] failed to satisfy the criteria for DSH" (p.311) were excluded. | "All attendances..." (p.311) were included. | No specific infor-mation reported, however, data for all eligible participants were included in analyses. | "The notes of the... episodes of DSH were then exam-ined..." (p.311). | Cases were identified from a "discharge diagnosis of 'self inflicted injury'" (p.311). Ascertain-ment of DSH from diagnostic or dis-charge codes alone has been shown to under-enumerate cases.^49^ | Hospital/medical records used. | No specific inform-ation reported, how-ever, it is likely that the same mode of data collection was used. | The study reported outcome(s) at pre-sentation and/or immediately post-discharge and so it is likely the length of the prevalence period was appro-priate. | There were no errors in the reporting of the numerator(s) and denominator(s) for the prevalence. |
| --- | --- | --- | --- | --- | --- | --- | --- | --- | --- | --- |
| **Dennis 2001** | An audit was undertaken of "...attendances aged 16 and over at the Lei-cester Royal Infirmary A&E department" (p.448). However, it is unclear whether this region is representative of the national population. | "...attendances aged 16 and over [were inc-luded]" (p.449). Only "...cases [were] ex-cluded if they were not episodes of DSH as defined by Morgan. (p.449). Additionally, "[i]f any patient left the department before, or during the A&E doctors' assessment they were excluded..." (p.449). | "All attendances...were identified" (p.448). | Whilst "[a] total of 1359 episodes of adult DSH were identified" (p.448), data for 1356 were included in subse-quent analyses, giv-ing an effective non-response rate of 0.3%. | Data were collected by "...examining the case notes and using the audit instrument to assess whether the information required had been recorded" (p.449). | "...attendances...with a diagnostic coding of 'self-inflicted inj-ury'...It is unlikely that episodes of DSH would have been given any other diagnostic coding" (p.449). Ascertain-ment of DSH from diagnostic or dis-charge codes alone has been shown to under-enumerate cases.^49^ | Hospital/medical records used. | No specific inform-ation reported, how-ever, it is likely the same mode of data collection was used for all participants. | The study reported outcome(s) at pres-entation and/or immediately post-discharge and so it is likely the length of the prevalence period was appro-priate. | Denominator for prev-alence of psychosocial assessment had to be reverse engineered as not clearly reported. Additionally, different denominators used in the tables and text with no explanation as to why |
| **Doshi 2005** | Data from the...[Nat-ional Hospital Ambu-latory Medical Care Survey] were combined to generate national estimates of ED visits... National estimates are obtained through use of assigned patient visit weights..." (p.369). | Whilst all "ED visits for attempted suicide and self-inflicted inj-ury...were included" (p.370), the sampling frame did exclude those presenting to "...federal, military, and Veterans Affairs hospitals, in the United States" (p.370). | The National Hospital Ambulatory Medical Care Survey uses "...a 4-stage probability sample...[and] samples from geographic prim-ary sampling units, hospitals within the primary sampling units, EDs within the hospitals, and patients within the EDs" (p.370). | No specific infor-mation reported, however, data for all eligible participants were included in analyses. | "Hospital staff collect data by medical record review..." (p.370). | "ED visits for attem-pted suicide and self-inflicted injury were defined by an Inter-national Classifi-cation of Diseases, Ninth Revision, Clinical Modifi-cation (ICD-9-CM) code of E950 to E959 in the primary diagnosis field." (p.370). Ascertain-ment of DSH from diagnostic or dis-charge codes alone has been shown to under-enumerate cases.^49^ | Hospital/medical records used. | No specific inform-ation reported, how-ever, it is likely the same mode of data collection was used for all participants. | The study reported outcome(s) at pre-sentation and/or immediately post-discharge and so it is likely the length of the prevalence period was appro-priate. | There were no errors in the reporting of the numerator(s) and denominator(s) for the prevalence. |
| **Ebbage 1994** | "Case notes were ex-amined from three Lon-don teaching hospitals in two district health auth-orities in North-East London" (p.515). How-ever, it is unclear whether these regions would be representative of the national population. | The sample "...compr-ised 34% of patients with self-poisoning seen in the A & E departments, a further 66% were admitted." (p.515). It would therefore appear that those who were ad-mitted to a ward were excluded. Addition-ally, those engaging in other methods would have been excluded. | "Three hundred case records were exam-ined: 150 from each health authority..." (p.515). However, no specific information on the method(s) used to select participants was reported. | No specific infor-mation reported, however, data for all eligible participants were included in analyses. | "Case notes were examined…" (p.515). | NR. | Hospital/medical records used. | No specific inform-ation reported, how-ever, it is likely the same mode of data collection was used for all participants. | The study reported outcome(s) at pre-sentation and/or immediately post-discharge and so it is likely the length of the prevalence period was appro-priate. | There were no errors in the reporting of the numerator(s) and denominator(s) for the prevalence. |
| **Ege 2022** | NR. | “Patients who [sic] referred to our emergency service because of suicide attempts and have a psychiatric consul-tation note between January 2015 and 2018 were included…” (p.2). Therefore, those engaging in DSH without suicidal intent were excluded. | NR. | NR | NR | NR | NR | NR | The study reported outcome(s) at pre-sentation and/or immediately post-discharge and so it is likely the length of the prevalence period was appro-priate. | There were no errors in the reporting of the numerator(s) and denominator(s) for the prevalence. |
| **Ferreira 2016** | Whilst the "...catchment area composed of 26 municipalities, contain-ing an estimated popu-lation of 1,300,000 in-habitants and located in the southeast of the country. Ribeirao Pretois the main city of the reg-ion, with 600,000 in-habitants and a per capita gross domestic product of approx-imately US$14,000.." (p.193), it is unclear whether this region is representative of the national population. | "...patients admitted to the emergency unit... with a diagnosis of 'intentional self-inflic-ted injury' and coded as X60 to X84, accord-ing to the tenth edition of International Class-ification of Diseases" (p.193) were included. There were no explicit exclusion criteria. | "The study population consisted of all patients..." (p.193). | No specific infor-mation reported, however, data for all eligible participants were included in analyses. | "Data were collected from medical records…" (p.193). | "...diagnosis of ‘‘in-tentional self-inflict-ed injury’’ and coded as X60 to X84, acc-ording to the tenth edition of Inter-national Classif-ication of Diseases (ICD10)" (p.193). Ascertainment of DSH from diagnostic or discharge codes alone has been shown to under-enumerate cases.^49^ | Hospital/medical records used. | No specific inform-ation reported, how-ever, it is likely the same mode of data collection was used for all participants. | The study reported outcome(s) at pre--sentation and/or immediately post-discharge and so it is likely the length of the prevalence period was appro-priate. | There were no errors in the reporting of the numerator(s) and denominator(s) for the prevalence. |
| **Ferreira 2019** | No specific information is reported from which hospitals participants were recruited. There-fore, it is difficult to determine whether this region is representative of the national population. | This study included only "...adolescents, ages 12 to 17" (p.318) who had engaged in intentional drug over-dose. Therefore, those who engaged in other methods of DSH would have been excluded. | NR | No specific infor-mation reported, however, data for all eligible participants were included in analyses. | Whilst some infor-mation was ascertain-ed "[t]hrough consul-tation of medical records..." (p.318), it is unclear whether information for the outcome(s) of rele-vance to this review was ascertained from participant self-report and/or hospital/ medical records. | NR | NR | No specific inform-ation reported, how-ever, it is likely the same mode of data collection was used for all participants. | The study reported outcome(s) at pre-sentation and/or immediately post-discharge and so it is likely the length of the prevalence period was appro-priate. | There were no errors in the reporting of the numerator(s) and denominator(s) for the prevalence. |

| **Gardner 1982** | Patients were recruited from "...the accident department of Adden-brooke's Hospital" (p.491). However, it is unclear whether the catchment area served by this hospital is representative of the national population. | Only "...patients with self-poisoning" (p.491) were included. There-fore, those engaging in other methods of DSH would have been excluded. | "…patients were con-secutively seen in the accident department… of whom 115 were dis-charged and the remainder admitted. Ninety-eight of the 199 admissions were randomly selected" (p.491) for inclusion, in addition to all 115 who were discharged. | No specific infor-mation reported, although we would note the data repor-ted on p.492 would not appear to be based on the grand denominator, sugg-esting there may have been some non-response. | NR | NR | NR | "Patients [that were discharged] were in-terviewed...in their homes whenever possible, about three weeks after their discharge from the accident department, and a questionnaire was completed. Pat-ients [that were admitted] were inter-viewed on the ward shortly after their admission using a similar question-naire" (p.491). How-ever, it is unclear how similar these questionnaires would have been. | "Patients were followed up at one year" (p.491). It is therefore likely that the length of the prevalence period was appropriate. | The percentages repor-ted on p.492 do not appear to be based on the total denominator, however, the appro-riate denominator has not been reported. |
| --- | --- | --- | --- | --- | --- | --- | --- | --- | --- | --- |
| **Geulayov 2016** | "This observational study was undertaken in the three centres of the Multicentre Study of Self-harm in England... [t]he three study centres include socioeconomic-ally diverse populations" (p.2). These centres have previously been shown to have good representa-tion of the national population. | "We included all in-dividuals aged 15 years and over who presented to five general hospital emergency depart-ments (EDs) following self-harm..." (p.2). And whilst "...the data of 25 individuals whose sex was un-known..." (p.2) were excluded, there were no systematic exclusions. | "Information on all episodes of self-harm was collected..." (p.2). | No specific infor-mation reported, however, data was missing for only 19.8% of cases. | Data were "collected in two ways: (1) completion of assess-ments (of mental state, risks and needs) by the general hos-pital psychiatric ser-vice (and in Manche-ster also by ED staff) and (2) scrutiny of ED electronic data-bases" (p.2). | No specific infor-mation reported. However, previous publications using data from the Multi-centre Study of Self-Harm have indicated that cases were ascertained follow-ing a review of hospital/medical records. | Hospital/medical records used. | No specific inform-ation reported, how-ever, previous studies using data from the Multicentre Study of Self-harm in England have indicated that different modes of data collection may be used for those who receive a psycho-social assessment in the ED and those who do not. | The study reported outcome(s) at pre-sentation and/or immediately post-discharge and so it is likely the length of the prevalence period was appro-priate. | There were no errors in the reporting of the numerator(s) and denominator(s) for the prevalence. |

| **Gunnell 2005** | "...hospitals were selec-ted within each of the 8 former Health Regions in England from four strata reflecting above or below median estimated self-harm admission and readmission rates" (p.67). Further, these hospitals were located in "...areas with varying levels of socioeconomic deprivation" (p.68). It is therefore likely the study is representative of the national population. | "Forms were comple-ted for all self-harm attendances [for those aged 18 and over] whether they were for overdoses, self-laceration or other methods" (p.68). | "...a stratified random sample of 32 hospitals was selected from a list of all general hos-pitals in England prov-iding an accident and emergency (A&E) service" (p.67). And whilst "[a]ll but one of the 32 hospitals initi-ally approached agreed to take part. The hos-pital that declined to participate was replac-ed by another random-ly selected hospital" (p.67). However, "...[d]ata from one participating hospital (n=189 episodes) are excluded from this analysis as they were provided in aggregate form" (p.68). Add-itionally, no specific information on the method(s) used to select participants was reported. | No specific infor-mation reported, however, data was missing for only 0.6% of cases. | NR | Whilst "...our def-inition of self-harm which was: 'a deli-berate non-fatal act whether physical, drug overdosage or poisoning, done in the knowledge that it was potentially harmful and in the case of drug over-dose that the amount taken was excess-ive'" (p.68), it is unclear whether this determination was made on the basis of diagnostic codes alone, or whether additional review was undertaken to investigate intent in ambiguous cases. | NR | "...[w]here individ-uals were identified as having been miss-ed from the audit, forms were comple-ted by trust staff using the subject's A&E, medical and mental health rec-ords. Similarly, these sources were used to obtain information where the audit forms had not been fully completed" (p.68). | The study reported outcome(s) at pre-sentation and/or immediately post-discharge and so it is likely the length of the prevalence period was appro-priate. | There were no errors in the reporting of the numerator(s) and denominator(s) for the prevalence. |
| --- | --- | --- | --- | --- | --- | --- | --- | --- | --- | --- |
| **Haq 2010** | No specific information is reported from which hospitals participants were recruited. There-fore, it is difficult to determine whether participants were recruited from a region representative of the national population. | NR | "Twenty-five sets of medical notes were collated at random for patients who had pres-ented with self harm to the emergency department" (p.1). | No specific infor-mation reported, however, data for all eligible participants were included in analyses. | "Notes were review-ed for evidence of... psychiatric assess-ment...and further referral" (p.1). | NR | Hospital/medical records used. | No specific inform-ation reported, how-ever, it is likely the same mode of data collection was used for all participants. | The study reported outcome(s) at pre-sentation and/or immediately post-discharge and so it is likely the length of the prevalence period was appro-priate. | There were no errors in the reporting of the numerator(s) and denominator(s) for the prevalence. |
| **Haw 2002** | Data were collected for those "...who presented to the district general hospital in Oxford..." (p.58). Data from the Oxford centre only of the Multicentre Study of Self-harm in England has previously been shown to not be representative of the national population. | "The subjects in this study were a sub-group...with a diag-nosis of depressive disorder [constituting] 70.7% of the original sample..." (p.58). | NR | "Eighty-six of the 106 (81.1%) depre-ssed patients were interviewed at follow-up...[how-ever, t]here was no difference between those followed up and those not, with respect to age, sex, previous episodes of DSH, severity of de-pression, or whether they were receiving psychiatric treatment at the time of the index DSH episode" (p.58). | No specific infor-mation reported, however, based on information reported in other studies using data from the Multi-centre Study for Self-harm in England it is likely data were col-lected from a mixture of hospital/medical records and partici-pant self-report (for those followed-up). | "The definition of DSH included acts of self-poisoning and self-injury, but ex-cluded self-cutting that was part of a repetitive pattern of self-mutilation" (p.58). This was identified from a dedicated sur-veillance system for self-harm. Previous work using this sys-tem has indicated cases are ascertained following scrutiny of medical/hospital records and patient self-report. | Hospital/medical records used. | No specific inform-ation reported, how-ever, previous work using data from the Oxford Monitoring System has indicated that different modes of data collection may be used for those who receive a psych-osocial assessment in the ED and those who do not. | "...we examined the psychiatric treatment these patients were receiving...at follow-up 12-20 months later" (p.58). With regards to follow-up, the majority of par-ticipants (79/86) were interviewed between 12-16 mon-ths. It is therefore likely that the length of the prevalence period was appro-priate. | There were no errors in the reporting of the numerator(s) and denominator(s) for the prevalence. |
| **Hawton 1992a** | The study setting was "...a general hospital in Oxford...The general hospital receives all re-ferred patients from Ox-ford area and the surr-ounding area" (p.1409). Data from the Oxford centre only of the Multi-centre Study of Self-harm in England has previously been shown to not be representative of the national population. | All "...people referred to the general hospital as a result of...deli-berate self poisoning or self injury" (p.1409) were included. There were no specific exclusion criteria. | Given that "[p]atients referred after self poisoning or self injury are identified by the monitoring system, irrespective of whether they are referred to the emergency psychiatric service in the hospital" (p.1409), it is likely a census could have been undertaken. | No specific infor-mation reported, however, data for all eligible participants were included in analyses. | Whilst "Those referr-ed…received a de-tailed psychosocial assessment…after which a data sheet was completed for entry to the com-puterised monitoring file" (p.1409), it is unclear whether in-formation for the outcomes of rele-vance to this review was ascertained from participant self-report and/or hospital/med-ical records. | No specific infor-mation reported, however, previous studies using data from the Multicentre Study of Self-harm has indicated cases were ascertained from scrutiny of hospital/medical records. | NR | "Those referred to the [psychiatric] service (83 2% [of the sam-ple]) received a de-tailed psychosocial assessment...Through scrutiny of accident and emergency de-partment records a limited amount of information was also available on patients referred to the hos-pital but not to the psychiatric service" (p.1409). Therefore, slightly different methods of data collection were used for 16.8% of the sample. | The study reported outcome(s) at pres-entation and/or immediately post-discharge and so it is likely the length of the prevalence period was appro-priate. | There were no errors in the reporting of the numerator(s) and denominator(s) for the prevalence. |
| **Hendrix 2013** | "The ED of our 1800 bed academic teaching hospital in Leuven, Bel-gium, has an annual cen-sus of approximately 55 000 patients..." (p.1), however, it is unclear whether this region is representative of the national population. | "All patients older than 16 years admitted to the ED with a his-tory of DSP were inc-luded...[and only d]ata on patients who pre-sented with isolated alcohol intoxication or with accidental intox-ication were excluded from the study" (p.1). | This study was a "...retrospective med-ical record review of all episodes of DSP presenting to the ED over a 12 month period" (p.1). | No specific infor-mation reported, however, data for all eligible participants were included in analyses. | "...data elements were extracted from the hospital infor-mation system for each registered patient [and included] final destination" (p.1). | "Selection of cases was made using physician discharge diagnosis codes that indicated any form of 'DSP' as a primary diagnosis" (p.1). As-certainment of DSH from diagnostic or discharge codes alone has been shown to under-enumerate cases.^49^ | Hospital/medical records used. | No specific inform-ation reported, how-ever, it is likely the same mode of data collection was used for all participants. | The study reported outcome(s) at pre-sentation and/or immediately post-discharge and so it is likely the length of the prevalence period was appro-priate. | There were no errors in the reporting of the numerator(s) and denominator(s) for the prevalence. |
| **Hengeveld 1988** | "This study included all patients referred to the UHL [University Hos-pital Leiden]..." (p.284). However, it is unclear whether this region is representative of the national population. | "...patients referred to a university hospital following an attempted suicide" (p.283) were included. There were no specific exclusion criteria. | NR | "...the response rate was actually 120/167 (72%)" (p.284). | "...patients who were asked to co-operate... were eventually inter-viewed" (p.284). Ad-ditionally, "...follow- up [interviews] by telephone" (p.284) were undertaken. | Whilst "...a suicide attempt was defined as: an intentional or well-considered self-inflicted injury or in-gestion of a sub-stance in a dose gen-erally regarded to be more than therapeu-tic, with the purpose of achieving certain desired changes through its actual or possible physical consequences" (p.284), it is unclear whether this determi-nation was made on the basis of diag-nostic codes alone, or whether additional review was under-taken to investigate intent in ambiguous cases. | Participant self-report | No specific inform-ation reported, how-ever, it is likely the same mode of data collection was used for all participants. | Participants com-pleted an "interview 4 to 6 weeks after discharge and with a follow-up interview by telephone about 6 months later (i.e., 7 months after dis-charge" (p.284). It is therefore likely the length of the prev-alence period was appropriate. | It is unclear what the correct denominator was for data presented in Table 4, p.286. We therefore had to estimate the correct data for this review. |
| **Joubert 2012** | "A pilot project aimed at examining the psycho-social precipitating fac-tors of people who had attempted suicide was conducted at Western Hospital..." (p.68). Furthermore, "...the loc-ation of the pilot project in western metropolitan Melbourne was signifi-cant as the region had the highest rate in Vic-toria of Years Living with a Disability (YLD) and Disability Adjusted Life Years(DALY) for all mental dis-orders for both men and women. The number of primary care physicians (PCPs) to population was among the lowest in Victoria..." (p.67). It is therefore likely this region is not representative of the national population. | "The files of patients who had presented due to attempted suicide... were data mined" (p.68). There were no explicit exclusion criteria. | Whilst "[i]nformation on patients presenting with suicidality was obtained from the hospital database by their hospital unit rec-ord number. There were 72 patients detected" (p.68), no specific information on the method(s) used to select participants was reported. | No specific infor-mation reported, however, data for all eligible participants were included in analyses. | Hospital/medical records "...were mined with an audit tool" (p.68). | NR. | Hospital/medical records used. | No specific inform-ation reported, how-ever, it is likely the same mode of data collection was used for all participants. | The study reported outcome(s) at pre-sentation and/or immediately post-discharge and so it is likely the length of the prevalence period was appro-priate. | There were no errors in the reporting of the numerator(s) and denominator(s) for the prevalence. |
| **Kang 2021** | "This retrospective study was conducted on pat-ients who visited the ED of the general hospital in a large urban city of South Korea...An annual average of 65,000 pat-ients visit this hospital, and more than 9600 patients are hospitalized each year" (pp.63-4). However, it is unclear whether this region is representative of the national population. | This study included "...patients [who] were referred to the ED following their self-harm behavior" (p.64). However, the study excluded "...64 pat-ients [for a number of reasons] including 9 cases who registrations were cancelled bec-ause their requests for care were refused..." (p.64). | "We referred all pa-tients who visited the ED following self-harm behavior to the responsible case management team" (p.64). | No specific infor-mation reported, however, data for all eligible participants were included in analyses. | "From the patients' EMR, we collected the following data... ED outcomes (ICU admission, general ward [GW] admiss-ion, and discharge)" (p.64). | NR | Hospital/medical records used. | "Patients who agreed to be managed by the case management team responded to all items on their forms. Meanwhile, the forms of those who did not agree were incom-plete; therefore, we had to obtain as much information as possible from their electronic medical records (EMR)" (p.64). | The study reported outcome(s) at presentation and/or immediately post-discharge and so it is likely the length of the prevalence period was appro-priate. | There were no errors in the reporting of the numerator(s) and denominator(s) for the prevalence. |

| **Kapur 2003** | Whilst "[t]he study was carried out in six hos-pitals in the North West of England. Hospitals A, B and C were teaching hospitals. Hospitals D, E and F were district gen-eral hospitals. The three teaching hospitals and one of the district hos-pitals (Hospital D) serv-ed predominantly urban, inner city catchment areas. Two district hos-pitals (Hospitals E and F) served mixed urban and rural catchment areas" (p.391), it is unclear where these hospitals are located, and therefore whether they are representative of the national population. | Whilst "...episodes of deliberate self-poison-ing involving patients over 16 years of age" (p.391) were included, episodes involving other methods of DSH would have been excluded. | "...all episodes..." (p.391) were included. | No specific infor-mation reported, however, data for all eligible participants were included in analyses. | "...data were retro-spectively...obtained from the Patient Ad-ministration System (PAS) in each hospital" (p.391). | "Episodes were inc-luded if they met the standard definition used in the WHO Multicentre Study on Parasuicide" (p.391). Additionally, "where possible we made use of existing ward and Accident and Emergency based information systems, such as referral led-gers and computer-ised databases. This information was sup-plemented by exam-ining Accident and Emergency notes of all patients (regard-less of presenting complaint) present-ing during the study period, in order to ensure we did not miss any episodes" (p.391). | Hospital/medical records used. | No specific inform-ation reported, how-ever, it is likely the same mode of data collection was used for all participants. | The study reported outcome(s) at pres-entation and/or immediately post-discharge and so it is likely the length of the prevalence period was appro-priate. | There were no errors in the reporting of the numerator(s) and denominator(s) for the prevalence. |
| --- | --- | --- | --- | --- | --- | --- | --- | --- | --- | --- |
| **Kapur 1999** | "The study was carried out in four teaching hospitals. Leeds General Infirmary and Manches-ter Royal Infirmary are inner city hospitals serv-ing populations of app-roximately 400 000 and 200 000, respectively. Leicester Royal Infirm-ary serves 700 000 people, including a sub-stantial rural population. The University Hospital in Nottingham has a predominantly urban catchment population of 800 000" (p.599). However, it is not clear whether these regions are representative of the national population. | "We included in the study...patients over 16 years of age who at-tended the hospitals with deliberate self-poisoning...[however w]e decided not to include cases of deliberate self-injury..." (p.599). | "We included in the study all patients..." (p.599). | No specific inform-ation reported, how-ever, data for all eligible participants were included in analyses. | "Where possible, we made use of existing ward and accident and emergency (A&E) based inform-ation systems, such as referral ledgers and computerised databases. This information was supplemented by examining A&E notes..." (p.600). | "Copies of specialist deliberate self-poi-soning assessments were...examined. All in-patient data were retrospectively checked against ad-mission and dis-charge information for deliberate self-poisoning (ICD codes X60-X69), obtained from the Patient Administra-tion System in each hospital" (p.600). | Hospital/medical records used. | No specific inform-ation reported, how-ever, it is likely the same mode of data collection was used for all participants. | The study reported outcome(s) at pres-entation and/or immediately post-discharge and so it is likely the length of the prevalence period was appro-priate. | There were no errors in the reporting of the numerator(s) and denominator(s) for the prevalence. |

| **Kawahara 2017** | "The eligible subjects were patients admitted to the ER of National Hos-pital Organization Kum-amoto Medical Center (NHO-KMC hospital), Japan...The NHO-KMC hospital is a tertiary medical facility and is one of the 284 govern-ment-designated emerg-ency critical care centers in Japan...[and] is the only facility in the city that is equipped with both an emergency critical care center and a psychiatry ward, cover-ing approximately 40% of ambulance transport-ation cases due to self-harm in Kumamoto prefecture (approx-imately 1.8 million inhabitants within an area of 7404 km2" (p.422). However, it is unclear whether this region is representative of the national population. | "...patients who pre-sented at an ER following self-harm" (p.422), and only "[t]hose who's [sic] self-harm was fatal (i.e. death by suicide) were excluded" (p.422). | "The eligible subjects were [all] patients admitted..." (p.422). | No specific infor-mation reported, however, data for all eligible participants were included in analyses. | No specific infor-mation reported. However, given that "...variables [that] were not well doc-umentted in most of the medical record" (p.422) were exclud-ed, it is likely that data were collected from medical records. | Whilst "[i]n our def-inition, self-harm included both self-harm and aborted or interrupted suicide attempts. Self-harm was defined as 'any act of self-poisoning or self-injury ire-spective of the app-arent purpose of the act'...or irrespective of level of medical seriousness" (p.422), it is unclear whether this determination was made on the basis of diagnostic codes alone, or whether additional review was under-taken to investigate intent in ambiguous cases. | Hospital/medical records used. | No specific inform-ation reported, how-ever, it is likely the same mode of data collection was used for all participants. | Given that "...[t]he subjects were followed-up for one year after the index episode" (p.422) it is likely the length of the prevalence period was appro-priate. | There were no errors in the reporting of the numerator(s) and denominator(s) for the prevalence. |
| --- | --- | --- | --- | --- | --- | --- | --- | --- | --- | --- |
| **Kinmond 2000** | Whilst "...individuals presenting with self-harm to one major hos-pital in the West Mid-lands" (p.271) were recruited, no specific information is reported from which hospitals participants were recruited. Therefore, it is difficult to determine whether participants were recruited from a region representative of the national population. | NR | NR | Whilst data for all eligible participants were included in analyses for the 1997 cohort, out-come(s) were not available for those in the 1990 cohort given the way in which data were recorded in this study. | No specific infor-mation reported, however, given than "[a]n audit of self-harm presentations to A&E, together with demographic data" (p.217) was under-taken, it is likely that data were collected from medical records. | Whilst "[t]he term 'self-harm' was used in this audit to inc-lude any form of self-inflicted harm, as in Kreitman's (1977) definition" (p.217), it is unclear whether this deter-mination was made on the basis of diag-nostic codes alone, or whether additional review was under-taken to investigate intent in ambiguous cases. | Hospital/medical records used. | Data "...were collect-ed during the first six months...of 1990. The audit was repea-ted in the first half of 1997" (p.217). Given the time lapse be-tween these two per-iods, it is possible different modes of data collection could have been used. | The study reported outcome(s) at pre-sentation and/or immediately post-discharge and so it is likely the length of the prevalence period was appro-priate. | There were no errors in the reporting of the numerator(s) and denominator(s) for the prevalence. |

| **Knipe 2021** | “Data were collected from the Teaching Hos-pital Peradeniya in the Kandy district in the Central Province of Sri Lanka. The Kandy district mirrors the demographic profile of the country in terms of the age and sex distri-bution…” (p.893). | “…cases of self-poisoning (ie, inten-tional self-harm by ingesting poison)” (p.894) were included. However, those engaging in other methods of DSH would have been excluded. | “…all self-poisoning cases admitted to the ward between Jan 1, 2019, and Aug 31, 2020” (p.894) were included. | No specific infor-mation reported, however, data for all eligible participants were included in analyses. | “Admission book data and information from bed head tickets were used to identify cases…” (p.894). | “Data were collected by trained clinical research assistants under the supervision of the study authors, who regularly check-ed the admission books against the data collected from bed head tickets to ensure cases were not missed. The data were extracted by a single research assistant.” (p.894). | Hospital/medical records used. | No specific inform-ation reported, how-ever, it is likely the same mode of data collection was used for all participants. | NR | There were no errors in the reporting of the numerator(s) and denominator(s) for the prevalence. |
| --- | --- | --- | --- | --- | --- | --- | --- | --- | --- | --- |
| **Lin 2014** | Participants were re-cruited from "...the ED of a general hospital in Taipei, Taiwan..." (p.318). However, it is not clear whether this region is representative of the national population. | "The inclusion criteria allowed all patients who had attempted suicide and self-harmers, but excluded patients with only suicidal ideation" (p.318). | "All participants were consecutively recruited..." (p.318). | No specific infor-mation reported, however, data for all eligible participants were included in analyses. | "All medical records in the ED, including emergency records, admission and dis-charge notes, prog-ress notes, consulta-tion sheets, SW [social work] notes, and nursing notes, were retrospectively reviewed" (p.318). | Whilst "'[s]uicidal behavior' was de-fined as any self-harm incident that was both brought to the attention of med-ical staff in the ED and linked with the patient's expressed intent to commit suicide or hurt him/ herself [and further authors] did not try to differentiate bet-ween 'real' suicide attempts and 'ges-tures' (manipulative, attention-seeking acts), because sui-cide attempts and self-harm behaviors were not mutually exclusive" (p.318), it is unclear whether this determination was made on the basis of diagnostic codes alone, or whether additional review was undertaken to investigate intent in ambiguous cases. | Hospital/medical records used. | No specific inform-ation reported, how-ever, it is likely the same mode of data collection was used for all participants. | NR | There were no errors in the reporting of the numerator(s) and denominator(s) for the prevalence. |

| **Marriott 2003** | "Leeds is one of the United Kingdom’s six largest cities. It is in the North of England and lies in poorest half of the UK on indices of social deprivation. Its age structure is closely similar to that of the UK as a whole" (p.646). It is therefore apparent that this region would not be representative of the national population. | "The final list of ep-isodes of self-harm comprised all those where the patient in-dicated that he or she had caused harm to himself or herself, or where a clinician was of the opinion that self-harm had occur-ed. Recreational use of drugs or alcohol was not included unless it was plain that an excess quantity of a recreational drug had been taken with suicidal intent" (p.646). | Whilst data for "...all patients aged 55 years or over who attended" (p.646) were eligible for inclusion, only data for "...a similar number drawn at random..." (p.646) were included. | The authors do note that "[t]he denomin-ators change slightly because there were a few attendances for which we could find no notes about the patient’s assessment and follow-up..." (p.647), however, missing data ranges from 1.1% to 2.2% (depending on outcome). | "As part of routine practice, accident and emergency reception staff routinely record, on each patient’s record card [inform-ation on treatment received]" (p.646). | "We examined the case-notes for every episode of care where self-harm was a likely reason for having attending the hospital. We were deliberately over-inclusive— exam-ining twice weekly all records at either hospital that were coded (whether at arrival transfer or discharge) as any of the following: deli-berate self-harm, overdose, poisoning, psychiatric, mental illness, behaving strangely, appears drunk, did not wait for examination, or left before treatment. Codes varied be-tween the two hos-pitals; this list in-corporates the two systems of computer-ised data. The search was later repeated twice, each time after a delay of several weeks — because computerised and manual records are amended some time after hospital attendance, and because some missing records take time to be returned to their proper place" (p.646). | Hospital/medical records used. | No specific inform-ation reported, how-ever, it is likely the same mode of data collection was used for all participants. | NR | There were no errors in the reporting of the numerator(s) and denominator(s) for the prevalence. |
| --- | --- | --- | --- | --- | --- | --- | --- | --- | --- | --- |
| **McCauley 2001** | "The 179-bed hospital, which has a psychiatric unit with 24 inpatient beds, is set in a rural county" (p.117). How-ever, no specific infor-mation is reported from which hospitals partici-pants were recruited. Therefore, it is difficult to determine whether participants were re-cruited from a region likely to be represent-tative of the national population. | "All cases of patients admitted after DSH to a rural general hos-pital...were identified" (p.116). There were no specific exclusion criteria. | "From the records identified, 100 were selected randomly for inclusion in the study using a random number table" (p.117). | No specific information reported, however, data for all eligible participants were included in analyses. | NR | Cases "...were ident-ified using ICD codes E950-R959 and E980-989..." (p.116). Ascertain-ment of DSH from diagnostic or dis-charge codes alone has been shown to under-enumerate cases.^49^ | NR | No specific inform-ation reported, how-ever, it is likely the same mode of data collection was used for all participants. | The study reported outcome(s) at pre-sentation and/or immediately post-discharge and so it is likely the length of the prevalence period was appro-priate. | There were no errors in the reporting of the numerator(s) and denominator(s) for the prevalence. |
| **McGill 2021** | "This study used a pro-spective longitudinal cohort...of index admiss-ions to the Calvary Mater Newcastle hos-pital for deliberate self-poisoning..." (p.3). Whilst "[i]t serves a primary referral urban population of over 410,000 (Newcastle, Lake Macquarie and Port Stephens) and is a tertiary referral centre for a further rural population of over 243,000 (Lower and Upper Hunter Valley)" (p.3), it is unclear whether this region is representative of the national population. | "Almost all poisoning presentations in the primary referral area are directed to the Calvary Mater New-castle ED..." (p.3). However, those engaging in other methods of DSH would have been excluded. | "Data were extracted for all index deliberate self-poisoning presentations" (p.3). | No specific infor-mation reported, however, data for all eligible participants were included in analyses. | "Standardised pre-formatted data col-lection sheets were used by clinicians to record information about participant... clinical variables... based on reporting from the patient, family, ambulance, pharmacist and treating doctors..." (p.3). | Whilst no specific information reported in this study, pre-vious studies using the HATS database has indicated cases are identified follow-ing a full review of hospital/medical records. | Hospital/medical records used. | No specific inform-ation reported, how-ever, it is likely the same mode of data collection was used for all participants. | The study reported outcome(s) at pre-sentation and/or immediately post-discharge and so it is likely the length of the prevalence period was appro-priate. | There were no errors in the reporting of the numerator(s) and denominator(s) for the prevalence. |
| **McGrath 1989** | "The study reviewed ret-rospectively the charts of all patients who were ad-mitted to the Princess Alexandra Hospital, Brisbane..." (p.318). However, it is unclear whether this region is representative of the national population. | "The patients who were included in the survey were those who intentionally had taken a greater-than-rec-ommended dose of medication" (p.318). Therefore, those en-gaging in other meth-ods of DSH would have been excluded. Additionally, "Patients who were admitted to hospital with iatro-genic drug toxicity were excluded from the survey. When the hospital admissions were thought to be as a result of recreational drug abuse rather than a result of deliberate self-poisoning these cases were excluded" (p.318). | "The study reviewed retrospectively the charts of all patients who were admitted... with a diagnosis of deliberate self-poisoning" (p.318). | The authors estimate that "...a small but uncertain number" (p.318) of presentat-ions were excluded because they ab-sconded from the ED. A further 15 were admitted to other hospitals as the ICU at the study site was at capacity, giving an effective non-response rate of 4.4%. | "The central casualty register was used as the source…" (p.318). | NR | Hospital/medical records used. | No specific inform-ation reported, how-ever, it is likely the same mode of data collection was used for all participants. | Whilst "[t]he follow-up of the patients could be deduced from the note that had been made in the charts" (p.318), no specific information on the length of the prevalence period was reported. | There were no errors in the reporting of the numerator(s) and denominator(s) for the prevalence. |
| **McNicholas 2011** | The study setting was "Ireland's largest nation-al paediatric teaching hospital (243 beds), which also has a catch-ment area covering the greater Dublin area" (p.192), however, it is unclear whether this region is representative of the national population. | "All children presen-ting to the hospital with DSH or suicidal ideation were ident-ified..." (p.191). There were no specific exclusion criteria. | The study included "all children and adoles-cents who presented with DSH" (p.192). | "In total, 231 child-ren with DSH and/or suicidal ideation (SI) presented to the hos-pital emergency de-partment...but 34 charts were missing from the archives" (p.192), giving an effective non-response rate of 14.7%. | Data "...were collect-ed from the clinical case-notes" (p.192). | NR. | Hospital/medical records used. | No specific inform-ation reported, how-ever, is likely the same mode of data collection was used for all participants. | The study reported outcome(s) at pres-entation and/or immediately post-discharge and so it is likely the length of the prevalence period was appro-priate. | There were no errors in the reporting of the numerator(s) and denominator(s) for the prevalence. |
| **Nakin 2007** | Whilst the study was conducted in "...two hos-pitals in a rural district of KwaZulu-Natal" (p.52), no specific infor-mation is reported from which hospitals partici-pants were recruited. Therefore, it is difficult to determine whether participants were re-cruited from a region likely to be represent-tative of the national population. | "This descriptive study included attempted-suicide patients ad-mitted to the two hospitals..." (p.53). There were no explicit exclusion criteria. | NR | "Of the 73 patients admitted during the study period, 59  (81%) were inter-viewed..." (p.53), giving an effective non-response rate of 19.0%. | "The first author... conducted interviews with each participant" (p.53). | NR | Participant self-report | No specific inform-ation reported, how-ever, it is likely the same mode of data collection was used for all participants. | Given that partici-pants were inter-viewed "...at least 3 months after admiss-ion" (p. 53) it is likely the length of the prevalence period was appro-priate. | There were no errors in the reporting of the numerator(s) and denominator(s) for the prevalence. |
| **Nordentoft 2005** | "In the Copenhagen Hospital Corporation, four emergency rooms and six intensive care units were chosen...at general hospitals..." (p.214). However, it is unclear whether this region is representative of the national population. | No specific inclusion criteria reported, how-ever, it is implied all presentations to either emergency depart-ments and/or intensive care units following a suicide attempt were eligible for inclusion. Only those "...patients with postal address outside the catchment area of the Copen-hagen Hospital Cor-poration were exclude-ed from these analyses" (p.214). | NR | No specific infor-mation reported, however, data for all eligible participants were included in analyses. | Researchers "...filled out a form with 29 items on the basis of information from case records" (p.214). | "First, we extracted electronically all ad-missions and con-tacts with Reason for Contact Codes 4 [for intentional self-harm]. We discover-ed that many con-tacts with suicide attempts were not coded [for intent-ional self-harm] therefore, we also... identif[ied] contacts with the following ICD-10 action diag-noses: T36-T65.9 (poisoning), T71.9 (strangulation), T75.1 (drowning), T90-T98.3 (compli-cation to poisoning or other external fac-tor), K71.1-K71.9 (toxic hepatic fail-ure), S50-S51.9 and S54-S56.8 (lesions of elbow, and lower and upper limb), S60-S61.9, S64-S66.9 and S69-S69.9 (lesions of wrist and hand). At the Fred-eriksberg Hospital emergency room, it was not possible to perform an electronic search, because data only existed on paper. In this unit, all case records for the study period were read" (p.214). There-fore, different meth-ods of case ascertain-ment were used at different sites. | Hospital/medical records used. | No specific inform-ation reported, how-ever, it is likely the same mode of data collection was used for all participants. | The follow-up period for admissions "...to psychiatric depart-ment or...contact with outpatient fac-ilities [was assessed] within 1 week" (p.214). However, given previous work that suggests average wait times for psych-iatric outpatient treatment are longer than this, it is likely this prevalence period may not have been sufficient. | There were no errors in the reporting of the numerator(s) and denominator(s) for the prevalence. |
| **Olfson 2012** | "The primary source of data was the 2006 Med-icaid Analytic Extract files from all 50 states and the District of Columbia...These files include data on the patient’s eligibility, demographics, and medical service and on prescription claims data from the more than 40 million beneficiaries enrolled in Medicaid nationwide" (p.81). | "...patients 21 to 64 years of age [with an] emergency department discharge for deli-berate self-harm" (p.81) were included. "...patients were...ex-cluded if they received services in an institute-ional residential set-ting. These settings included prisons and correctional facilities, assisted living fac-ilities, group homes, nursing and custodial care facilities, hos-pices, and residential care facilities. Because patients who have fre-quent emergency de-partment visits for deliberate self-harm may be well known to emergency department staff and therefore not fully evaluated at each visit, we excluded emergency department visits for deliberate self-harm that were preceded by another such visit in the prior 30 days" (p.81). How-ever, data were only reported for those discharged to the community. Addition-ally, those not eligible for Medicaid would have been excluded. | Given that "[i]n our study, we examined the Medicaid medical service and pharmacy claims of patients 21 to 64 years" (p.81), it is likely a census could have been undertaken. | Outcome(s) are re-ported on those dis-charged to the community only, giving an effective non-response rate of 37.5% | "The primary source of data was the 2006 Medicaid Analytic Extract files..." (p.81). | "deliberate self-harm [was ascertained from] (International Classification of Diseases, Ninth Revision, Clinical Modification [ICD-9-CM] codes E950-E959) in any pos-ition on the claim" (p.81). Ascertain-ment of DSH from ICD codes alone has been shown to under-enumerate cases.^49^ | Administrative records used. | No specific inform-ation reported, how-ever, it is likely the same mode of data collection was used for all participants. | Outcome(s) were assessed "...within 30 days of emerg-ency department discharge" (p.81). However, given previous work that suggests average wait times for psychiatric out-patient treatment are longer than this, it is likely this prevalence period may not have been sufficient. | The denominator for some outcomes does not add up to what it should based on the total at baseline, the reasons for these discrepancies are unclear. |

| **Opmeer 2017** | The "[s]etting [was a] large hospital in South West England" (p.1). However, there is no specific information on the location. Previous work, however, clarifies that the database is maintained in the Em-ergency Departments of the Bristol Royal Infirm-ary, part of University Hospitals Bristol NHS Foundation Trust (UHBT) since 2010, and Southmead Hospital (initially Fenchay Hospital, prior to the transfer of acute services from Frenchay to Southmead), North Bristol NHS Trust (NBT) since 2013.^50^ Nevertheless, it is unclear whether this region is representative of the national population. | "...patients presenting to the ED following self-harm before the operating hours of LPS were extended...[and] after the extended LPS was fully operational " (p.2) were included. There were no specific exclusion criteria. | "Patients were ident-ified from the local Self-Harm Sur-veillance Register (SHSR). The SHSR was established in 2010 and records clinical and socio-demographic details of all hospital-pre-sentations for self-harm at the hospital" (p.2). | "Audits reveal SHSR [Self-Harm Surveillance Register] case ascertainment is >95%" (p.2). | No specific infor-mation reported, however, given the study design it is likely data were collected from hospital/medical records. | No specific infor-mation reported, however, previous work has indicated that all cases of DSH irrespective of sui-cidal intent are in-cluded in this system and that cases are ascertained follow-ing a review of hospital/medical records.^[[50]](#footnote-50)^ | NR | No specific inform-ation reported, how-ever, it is likely the same mode of data collection was used for all participants. | NR | There are errors in the numerators and denominators and proportions reported in Table 1. |
| --- | --- | --- | --- | --- | --- | --- | --- | --- | --- | --- |
| **Owens 1988** | Whilst "[p]atients from a wide area present at the A&E department..." (p.831) were recruited, it is unclear whether this region would be repre-sentative of the national population. | Whilst "[t]his study in-cluded all such pat-ients [i.e., those att-ending the emergency department following an episode of DSP] presenting...to the Nottingham A&E department" (p.831), "[t]hose few cases that were not diagnosed as deliberate self-poison-ing by the A&E doc-tor, or were of patients aged under 14, were excluded" (p.831). | "This study included all...patients..." (p.831). | No specific inform-ation reported, how-ever, data for all eligible participants were included in analyses. | Data was extracted "...from the booking-in ledgers used by the clerks, and.the A&E record for each patient" (p.831). | NR | Administrative records used | No specific inform-ation reported, how-ever, it is likely the same mode of data collection was used for all participants. | Given that partici-pants were followed over "...a 2-year follow-up period" (p.831), it is likely the length of the pre-valence period was appropriate. | There were no errors in the reporting of the numerator(s) and denominator(s) for the prevalence. |
| **Pang 1996** | "We have...undertaken a joint audit involving the Psychological Medicine Unit and the Accident & Emergency Department at a central London tea-ching hospital" (p.208). However, it is not clear whether this region is representative of the national population. | "...individuals referred to the A&E depart-ment following an episode of DSH...were identified and collec-ted" (p.208). There were no specific exclusion criteria. | "Case-notes for all individuals referred to the A&E department following an episode of DSH...were ident-ified and collected" (p.208). | No specific inform-ation reported, how-ever, data for all eligible participants were included in analyses. | Data were ascertain-ed from hospital/ medical records. Additionally, " [c]ase -notes for patients ad-mitted to the medical ward but not refer-red to the...Unit were traced to check whether they had been assessed by the on-call psychiatrists" (pp.208-9). | NR | Administrative records used | No specific inform-ation reported, how-ever, it is likely the same mode of data collection was used for all participants. | The study reported outcome(s) at pres-entation and/or immediately post-discharge and so it is likely the length of the prevalence period was appro-priate. | There were no errors in the reporting of the numerator(s) and denominator(s) for the prevalence. |
| **Pavarin 2014** | "The aims of this study are to describe the characteristics of admission for suicide-related presenting complaints in EDs of the metropolitan area of Bologna...[t]he metropolitan area of Bologna is a densely populated territory in the Emilia Romagna region, situated in northeastern Italy. It is made up of 50 municipalities...with a population of over 850,000 inhabitants" (p.407). However, it is unclear whether this region would be representative of the national population. | "All patients admitted...to the EDs of the metropolitan area of Bologna (excluding Sant’Orsola Hospital), northern Italy, for attempted suicide were retrospectively recruited" (p.407). Only those "... patients who died in hospital (17 cases) or in the ED (10 cases)..." (p.407) were excluded. How-ever, it is unclear whether the exclusion of participants pres-enting to the Sant’ Orsola Hospital would have negatively affect-ed the generalisability of this cohort. | "We examined the data on admissions for [all] attempted suicides in seven EDs for subjects…" (p.407). | No specific information reported, however, data for all eligible participants were included in analyses. | "All the records collected from each ED (including pre-hospital care reports, nurse notes, and physician records) were selected..." (p.407). | "Attempted suicide was defined as 'a potentially self-injurious behaviour with a nonfatal outcome, for which there is evidence that the person had the intent to kill him/her-self, but failed, was rescued or thwarted, or changed one’s mind'...For the case selection, we used electronic searches of admission databases and subsequent content analysis" (p.407). | Administrative records used | No specific information reported, however, given the study design it is likely the same mode of data collection was used for all participants. | The study reported outcome(s) at presentation and/or immediately post-discharge and so it is likely the length of the prevalence period was appro-priate. | There were no errors in the reporting of the numerator(s) and denominator(s) for the prevalence. |
| **Perquier 2017** | "The study took place in the Bichat-Claude Ber-nard University Hospital Centre, located in the north of Paris, France" (p.143). However, it is unclear whether this region is representative of the national population. | "All suicide attempt-ers, except those who need surgical or inten-sive care due to the severity of their att-empt..." (p.143) were eligible for partici-pation, however, those that "...were under 15 of age, refused to par-ticipate, were notable to understand study procedures or could not be interviewed due to severe cognitive or medical conditions" (p.143) were excluded. Whilst "[c]ompared to those who were not included, participants did not differ accord-ing to gender...or mean age..." (p.143), there may be other relevant prognostic factors that were not measured that did differ between those included and those excluded. | "Out of the 355 suicide attempters referred to the unit...69 patients were not included for no specific reason" (p.143). | No specific inform-ation reported, how-ever, data were mis-sing for 2.4% of cases. | Data were collected using a standard ass-essment form "filled out during a clinical interview" (p.143). | Whilst "[a] suicide attempt was defined as any 'act with a non-fatal outcome, in which an individual deliberately initiates a non-habitual be-havior that without intervention from others will cause self-harm, or deli-berately ingests a substance in excess of the prescribed or generally recognized therapeutic dosage', according to the WHO definition..." (p.143), it is unclear whether this deter-mination was made on the basis of diag-nostic codes alone, or whether additional review was under-taken to investigate intent in ambiguous cases. | NR | No specific inform-ation reported, how-ever, it is likely the same mode of data collection was used for all participants. | The study reported outcome(s) at pres-entation and/or immediately post-discharge and so it is likely the length of the prevalence period was appro-priate. | The proportions re-ported in Table 1, p.145 do not appear to be based either on the total number of partici-pants included at base-line, or the revised number provided within the table notes. |

| **Reith 2004** | "Subjects were identified from a clinical database maintained by the Hun-ter Area Toxicology Service (HATS)..." (p.521). However, it is unclear whether this region is representative of the national population. | "All admissions with deliberate or recreat-ional self-poison-ing...were selected" (p.521). However, "Transgender [persons] were exclud-ed, as were patients who died prior to being discharged from hospital during their index admission" (p.521). | NR | Data were extracted from psychosocial assessments, and "[f]ormal psychiatric assessment was per-formed on...3347 (91%) of the deli-berate self-poison-ings..." (p.521), giving an effective non-response rate of 9.0%. | Data were drawn "...from a clinical database maintained by the Hunter Area Toxicological Ser-vice (HATS)..." (p.521). | No specific inform-ation reported. How-ever, previous work using the Hunter Area Toxicological Service (HATS) has indicated that DSP is ascertained follow-ing a clinical review. | Hospital/medical records used. | No specific inform-ation reported, how-ever, it is likely the same mode of data collection was used for all participants. | The study reported outcome(s) at pres-entation and/or immediately post-discharge and so it is likely the length of the prevalence period was appro-priate. | There were no errors in the reporting of the numerator(s) and denominator(s) for the prevalence. |
| --- | --- | --- | --- | --- | --- | --- | --- | --- | --- | --- |
| **Runeson 2000** | The study included ad-missions "...to emerg-ency wards at Huddinge University Hospital, Padua University Hos-pital, and various centers affiliated to these two hospitals" (p.433), and whilst "[t]he catchment areas of the two hos-pitals are similar in size" (p.433), it is unclear why these two hospitals were chosen and whether the communities they serve are representative. | ...all suicide attempts admitted to emergency wards…" (p.433) were included. There were no explicit exclusion criteria. | The samples were collected consecut-ively…" (p.433). | No specific inform-ation reported, how-ever, data were mis-sing for 39.6% of cases. | No specific information reported. | Whilst the study identified "para-suicidal acts...in accordance with the following definition of such an act from the WHO/Eurostudy group…" (p.433), it is unclear whether this determination was made on the basis of diagnostic codes alone, or whether additional review was under-taken to investigate intent in ambiguous cases. | NR | No specific inform-ation reported, how-ever, it is likely the same mode of data collection was used for all participants. | The study reported outcome(s) at pres-entation and/or immediately post-discharge and so it is likely the length of the prevalence period was appro-priate. | There were no errors in the reporting of the numerator(s) and denominator(s) for the prevalence. |
| **Runeson 2001** | "Skaraborg County Hos-pital covers 75% of the catchment area of Skara-borg, including 280 000 inhabitants" (p.319), however, it is unclear whether this region is representative of the national population. | "All patients applying for medical help at the hospital...were includ-ed" (p.319). There were no explicit exclusion criteria. | "All patients applying for medical help at the hospital...were includ-ed which yielded a consecutive sample..." (p.319). | No specific inform-ation reported, how-ever, data for all eligible participants were included in analyses. | "Information was derived from medical records and, in the case of the study group, also by interviews" (p.320). | Whilst "...in accord-ance with the WHO definition of para-suicide..." (pp.319-20), it is unclear whether this deter-mination was made on the basis of diag-nostic codes alone, or whether additional review was under-taken to investigate intent in ambiguous cases. | Administrative records used | No specific inform-ation reported, how-ever, it is likely the same mode of data collection was used for all participants. | NR. | There were no errors in the reporting of the numerator(s) and denominator(s) for the prevalence. |

| **Russell 2010** | "Bradford is England's tenth largest city with a population of over 300, 000. Nearly half a million people in the Bradford Metropolitan District are served by a single acute hospital Trust with just one Emergency Department" (p.212), however, it is unclear whether this region is representative of the national population. | Those who received "...psychosocial ass-essment[s] following self-harm" (p.212) were included. "We then excluded four categories of patients from the study: those not assessed (usually because the patient was not fit for inter-view, n=19); those in whom self-harm was plainly unintentional (n=4); clerical errors, including duplicate entries and assess-ments entered in the wrong year (n=8); and those patients who had been assessed in the emergency department and whose assessment had been erroneously filed with the inpatient assessments (n=49)" (p.212). | "We obtained from the mental health service a computerized list of what was deemed to be all episodes of psych-osocial assessment following self-harm" (p.212). | "...45 assessments that, according to the computer records, should have been present were in fact missing...examin-ation of the relevant notes recovered 21 of the missing ass-essment sheets. This gave us a total of 867 assessments available for scru-tiny out of a possible 891, a retrieval rate of 97%" (p.212). | "Records were scrutinized..." (p.212). | Whilst "[s]elf-harm was defined in the conventional way as intentional self-poisoning or self-injury, irrespective of motivation (and excludes hospital attendances due to intoxication by alcohol or drugs)..." (p.212), it is unclear whether this deter-mination was made on the basis of diag-nostic codes alone, or whether additional review was under-taken to investigate intent in ambiguous cases. | Administrative records used | No specific inform-ation reported, how-ever, it is likely the same mode of data collection was used for all participants. | The study reported outcome(s) at pres-entation and/or immediately post-discharge and so it is likely the length of the prevalence period was appro-priate. | There were no errors in the reporting of the numerator(s) and denominator(s) for the prevalence. |
| --- | --- | --- | --- | --- | --- | --- | --- | --- | --- | --- |
| **Rygnestad 1991** | "In 1978 the Regional Hospital of Trondheim, Norway served a local population of about 200 000, including 153 000 aged over 13. Ten years later the numbers were 208 000 and 175 000, respectively" (p.53). However, it is unclear whether this region is representative of the national population. | "The patients included in the study were per-sons who deliberately had taken a drug or poison with the intent-ion to cause self-injury or in such an amount that they needed treat-ment in hospital" (p.53). Therefore, those engaging in other methods of DSH would have been excluded. | NR | No specific infor-mation reported, however, data for all eligible participants were included in analyses. | "All patients were in-terviewed by the same person, who also carried out the follow-up study and filled out the quest-ionnaires containing the variables of interest" (p.54). | Whilst "[t]he patients included in the study were persons who deliberately had taken a drug or poison with the intention to cause self-injury or in such an amount that they needed treatment in hospital" (p.53), it is unclear whether this determination was made on the basis of diagnostic codes alone, or whether additional review was undertaken to investigate intent in ambiguous cases. | It is unclear whether the questionnaire developed for this subject had been evaluated for reliability and validity. | No specific inform-ation reported, how-ever, it is likely the same mode of data collection was used for all participants. | The study reported outcome(s) at pres-entation and/or immediately post-discharge and so it is likely the length of the prevalence period was appro-priate. | There were no errors in the reporting of the numerator(s) and denominator(s) for the prevalence. |
| **Scott 1993** | Whilst "[t]his study is part of a larger project concerning...cases seen at the A&E Department of Dumfries and Gall-oway Royal Infirmary in 1988" (p.101) it is un-clear whether this region is representative of the national population. | "...self-poisoning and self-mutilation cases" (p. 101) were included. "One case had a diag-nosis of severe mental handicap [sic] with nu-merous attendances for self-mutilation. As this study was concerning adolescents of normal intelligence and inclu-sion could have biased data regarding prev-ious A&E attendance, this case was not included" (p.102). | NR | No specific inform-ation reported, how-ever, data for all eligible participants were included in analyses. | "Evidence of a ref-erral for psychiatric assessment was sought from medical and psychiatric notes" (p.102). | "Index cases of self-mutilation were id-entified from the Casualty Register. For any registered injury arousing sus-picion of self-muti-lation, e.g., lacer-ations to wrists, hands, or neck, these case notes were re-viewed for further details to ascertain whether or not injuries were self-inflicted" (p.101). | Hospital/medical records used. | No specific inform-ation reported, how-ever, it is likely the same mode of data collection was used for all participants. | The study reported outcome(s) at pre-sentation and/or immediately post-discharge and so it is likely the length of the prevalence period was appro-priate. | There were no errors in the reporting of the numerator(s) and denominator(s) for the prevalence. |
| **Shahid 2009** | Whilst the "...Aga Khan University Hospital... does not have a defined catchment area and pat-ients present from all over the city as well as from outside the city" (p.86), it is unclear whether this region is representative of the national population. | "...patients who pre-sented to the ED...over a period of 12 months" (p.85) were included. Data for those engag-ing in DSH were in-cluded, and no explicit exclusion criteria were reported. | "Medical records of all patients who presented to the ED..." (p.86) were included. | No specific inform-ation reported, how-ever, data were missing for 5.1% of cases. | Whilst "To record details of patients presenting with DSH, a special data extract-ion form was de-vised" (p.86), it is unclear whether this was based on inform-ation from participant self-report and/or hospital/medical records. | NR | NR | No specific inform-ation reported, how-ever, it is likely the same mode of data collection was used for all participants. | The study reported outcome(s) at pre-sentation and/or immediately post-discharge and so it is likely the length of the prevalence period was appro-priate. | There were no errors in the reporting of the numerator(s) and denominator(s) for the prevalence |
| **Shekunov 2021** | The study included "...all patients ages 0-18 in a single county [Olmstead County, Minnesota, USA] who were evalu-ated and treated for ex-cessive acetaminophen exposure" (p.758). It is not clear, however, whether the population in this county is rep-resentative of the nat-ional population. Add-itionally, the authors only included "...data from community mem-bers who have agreed to share medical records for research [via the Rochester Epidemiology Project]" (p.759). | Only those "evaluated for excessive acet-aminophen exposure" (p.759) were included. Therefore, those en-gaging in other meth-ods of DSH would have been excluded. | All patients who had previously "... agreed to share medical re-cords for research [via the Rochester Epi-demiology Project]" (p.759) were included. | No specific inform-ation reported, how-ever, data for all eligible participants were included in analyses. | "We extracted information from the electronic medical record…" (p.759). | "Data on overdose intentionality...were based on provider documentation from the ED evaluation. For patients who received psychiatric consultation, the des-cription contained in the psychiatrist’s note was used to determine intention-ality; for those not receiving a consul-tation, the ED phys-ician’s note was the data source. When intentionality was documented as am-biguous or ambiv  alent, the overdose was classified as intentional" (p.759). | Hospital/medical records used. | No specific inform-ation reported, how-ever, it is likely the same mode of data collection was used for all participants. | The study reported outcome(s) at pre-sentation and/or immediately post-discharge and so it is likely the length of the prevalence period was appro-priate. | There were no errors in the reporting of the numerator(s) and denominator(s) for the prevalence. |
| **Shuchman 1996** | The retrospective study was conducted a San Francisco General Hos-pital, a level 1 trauma center (p.755), however, it is not clear if this region is representative of the national population. | "All patients under 18 years old who were hospitalized…after att-empting suicide" (p.755) were included. "We excluded youths treated and released from the emergency department, those admitted solely for injuries for which psychosocial inter-vention was mandated..." (p.755). | ...all patients...admitted that year after attempt-ing suicide" (p.755) were included. | No specific inform-ation reported, how-ever, data for all eligible participants were included in analyses. | "Medical records were reviewed for... medical care in the hospital" (p.755). | No specific inform-ation reported, how-ever, as "[p]atients were identified through the logbook of the pediatrics de-partment and the trauma registry of the surgery depart-ment (p.755), it is likely cases were identified following review of hospital records. | Hospital/medical records used. | No specific inform-ation reported, how-ever, given the nature of the study it is likely the same mode of data collection was used for all participants. | The study reported outcome(s) at pre-sentation and/or immediately post-discharge and so it is likely the length of the prevalence period was appro-priate. | There were no errors in the reporting of the numerator(s) AND denominator(s) for the prevalence. |
| **Song 2012** | "We implemented an ED-based in-depth sur-veillance program for suicidal patients at two academic EDs in a met-ropolitan area" (p.923). However, it is unclear whether this region is representative of the national population. | "All adult patients older than 18 years who visited the ED for suicidal attempt were included" (p.923). There were no explicit exclusion criteria. | "All adult patients old-er than 18 years who visited the ED for suicidal attempt were included." (p.923). | No specific inform-ation reported, how-ever, data for all eligible participants were included in analyses. | "Physicians and psy-chiatrists conducted face-to-face inter-views and collected clinical information" (p.923). | NR | Participant self-report | No specific inform-ation reported, how-ever, it is likely the same mode of data collection was used for all participants. | The study reported outcome(s) at pre-sentation and/or immediately post-discharge and so it is likely the length of the prevalence period was appro-priate. | Data on the prevalence of psychiatric out-patient treatment re-ceived in months' 1 to 3 post-discharge could not be extracted and included in this review given the way in which it was reported (i.e., denominator data was incomplete). |
| **Suominen 2000** | Data were drawn from "...consecutive cases of attempted suicide refer-red to the general hos-pitals in Helsinki..." (p.119), however, it is unclear whether this region is representative of the national population. | "...consecutive cases of attempted suicide ref-erred to the general hospitals" (p.119) were included. Only "[s]imple alcohol intoxications were excluded" (p.119). | Whilst a "...sample of 114 patients aged 15 years or over taken from consecutive cases of attempted suicide" (p.119) was included, no specific information on the method(s) used to select participants was reported. | No specific inform-ation reported, how-ever, data for all eligible participants were included in analyses. | "...interviews were conducted according to a structured sch-edule, the European Parasuicide Study Interview Schedule I (EPSIS I)" (p.119), in addition to review of "...medical and psy-chiatric records..." (p.120). | Whilst "WHO's def-inition of parasuicide was applied…[and a]ttempted suicide and parasuicide are here used as synon-yms" (p.119), it is unclear whether this determination was made on the basis of diagnostic codes alone, or whether additional review was undertaken to investigate intent in ambiguous cases. | European Paras-uicide Study Inter-view Schedule I and hospital/medical records. | No specific inform-ation reported, how-ever, given the study design it is likely the same mode of data collection was used for all participants. | The study reported outcome(s) at pre-sentation and/or immediately post-discharge and so it is likely the length of the prevalence period was appro-priate. | There were no errors in the reporting of the numerator(s) and denominator(s) for the prevalence. |
| **Suominen 2004a** | "... data have been gath-ered from all four of the city's general hospitals treating deliberate self-harm patients" (p.721). However, it is unclear whether this region is representative of the national population. | "The data for the pre-sent study included all suicide attempts of Helsinki residents aged 15 years or more admitted to healthcare during the study period" (p.721). | "All consecutive deli-berate self-harm patients...were identified" (p.721). | "The data on treat-ment received were incomplete because of wrong identity numbers in 12 cases, and these were ex-cluded from the analyses" (p.721), giving an effective non-response rate of 1.0%. | "The data were gath-ered from case files..." (p.721). | Whilst "[t]he WHO definition of para-suicide was applied... and attempted sui-cide, deliberate self-harm and parasuicide used as synonyms" (p.721), it is unclear whether this deter-mination was made on the basis of diag-nostic codes alone, or whether additional review was under-taken to investigate intent in ambiguous cases. | Hospital/medical records used. | No specific inform-ation reported, how-ever, given the study design it is likely the same mode of data collection was used for all participants. | Given that "...we gathered record data on all health-care contacts...12 months after the index att-empt..." (p.721) it is likely the length of the prevalence per-iod was appropriate. | There were no errors in the reporting of the numerator(s) and denominator(s) for the prevalence. |

| **Sztajnkrycer 2007** | "...patients presenting to the Saint Mary's Hos-pital emergency depart-ment" (p.500) were re-cruited. However, it is unclear whether this region is representative of the national population. | "Inclusion criteria... included asymptomatic adult patients (defined at our institution as age 15years or older) who presented after known or suspected potentially toxic deliberate ingestion... [e]xclusion criteria included: [l]ack of history consistent with intoxication as the eti-ology for the clinical condition...[i]solated ethanol intoxication... [i]ngestion of sustain-ed release prepar-ations...[c]hronic drug intoxication, e.g., lith-ium, salicylates, dig-oxin...[p]resence of elevated drug level requiring prolonged medical therapy, e.g.. elevated acetamin-ophen, lithium, salicy-late levels...[p]resence of end-organ toxicity upon arrival necessi-tating prolonged med-ical care, e.g., elevated liver function tests in acetaminophen in-gestion...[p]resence of persistent self-injur-ious or violent behav-iors during the initial emergency department evaluation, posing a serious threat to safety of the patient, nursing, and ancillary staff... [p]resence of high-risk criteria" (p.500). Add-itionally, those engag-ing in other methods of DSH, such as self-poisoning and self-injury, would have been excluded. | "...all patients present-ing...with a complaint of over-dose or intoxication" (p.500) were included. | No specific inform-ation reported, how-ever, data for all eligible participants were included in analyses. | "...data was retro-spectively deter-mined via institut-ional sentinel event records...and Mayo Clinic Security records" (p.500). | Whilst "[d]eliberate ingestion was defin-ed as ingestion per-formed with the intent of causing self-harm or death" (p.500), it is unclear whether this deter-mination was made on the basis of diag-nostic codes alone, or whether additional review was under-taken to investigate intent in ambiguous cases. | Hospital/medical records used. | No specific inform-ation reported, how-ever, it is likely the same mode of data collection was used for all participants. | The study reported outcome(s) at pre-sentation and/or immediately post-discharge and so it is likely the length of the prevalence period was appro-priate. | There were no errors in the reporting of the numerator(s) and denominator(s) for the prevalence. |
| --- | --- | --- | --- | --- | --- | --- | --- | --- | --- | --- |

| **Tountas 2001** | Whilst "[t]his study inc-ludes [those] in the... Regional General Hos-pital of Nikea..." (p.2), it is not clear if this region is representative of the national population. | "This study includes all cases of drug intox-ication…" (p.2). Only "[p]atients taking any toxical or pharmaceut-ical substance by acc-ident or through ignor-ance were excluded..." (p.2). However, whilst there were no further explicit exclusion criteria, those engag-ing in other methods of DSH would have been excluded. | "This study includes all cases..." (p.2). | No specific inform-ation reported, how-ever, data for all eligible participants were included in analyses. | NR | "...the diagnosis of drug intoxication was based on in-formation provided by the patient him-self or by his/her closest relatives" (p.2). However, as there is significant stigma against att-emptted suicide, it is possible cases were significantly under-reported. | NR | No specific inform-ation reported; how-ever, it is likely that the same mode of data collection was used for collecting information from the participants. | The study reported outcome(s) at pres-entation and/or immediately post-discharge and so it is likely the length of the prevalence period was appro-priate. | There were no errors in the reporting of the numerator(s) and denominator(s) for the prevalence. |
| --- | --- | --- | --- | --- | --- | --- | --- | --- | --- | --- |
| **Vidalis 1987** | Whilst "All patients en-tering the Casualty De-partment..." (p.312) were recruited, no specific in-formation is reported from which hospitals participants were re-cruited. Therefore, it is difficult to determine whether participants were recruited from a region likely to be representative of the national population. | "...all admissions de-signated as over-doses..." (p.312) were included, however, some otherwise elig-ible participants "were found to be misdiag-nosed on first assess-ment...[and others were] excluded owing to a lack of inform-ation" (p.313). | NR | No specific inform-ation reported, how-ever, data for all eligible participants were included in analyses. | "We...studied the cas-ualty clinical notes of these patients... (p.312). | Whilst "[w]e search-ed the register for all admissions desig-nated as overdoses" (p.312), it is unclear whether this deter-mination was made on the basis of diag-nostic codes alone, or whether additional review was under-taken to investigate intent in ambiguous cases. | Hospital/medical records used. | No specific inform-ation reported, how-ever, it is likely the mode of data collection was the same for all participants. | NR | There were no errors in the reporting of the numerator(s) and denominator(s) for the prevalence. |
| **Whyte 2001** | The study was a "...case note audit [of all con-secutive]...assessments at Kettering general hos-pital" (p.98); however, it is unclear whether this region is representative of the national population. | "...consecutive sets of notes for DSH patients presenting to A & E from March 1999..." (p.99) were included. Whilst "[f]our patients' case notes were un-traceable" (p.99) and were excluded from analysis, this is un-likely to have signify-cantly compromised generalisability. | NR | No specific inform-ation reported, how-ever, data for all eligible participants were included in analyses. | NR | Whilst "[c]ases were identified using the A & E computerised patient database" (p.99), it is unclear whether this deter-mination was made on the basis of diag-nostic codes alone, or whether additional review was under-taken to investigate intent in ambiguous cases. | NR | No specific inform-ation reported, how-ever, it is likely the same mode of data collection was used for all participants. | The study reported outcome(s) at pre-sentation and/or immediately post-discharge and so it is likely the length of the prevalence period was appro-priate. | There were no errors in the reporting of the numerator(s) and denominator(s) for the prevalence. |

| **Witt 2023** | Study setting was “…the Royal Melbourne Hos-pital ED…The Royal Melbourne Hospital is one of the largest and busiest metropolitian adult-treating public EDs in the region. This site serves a primary catchment area of approximately 1,554,029 people and receives over 78,600 presentations to the ED each year. The catchment area includes one of Melbourne’s fastest growing growth corridors. It is largely representative of the broader Australian population in terms of age and sex but has a higher-than-average population of those born outside of Australia, and a lower-than-average population of Aboriginal and Torres Strait Islander people” (p.2). | “Each [hospital] record was…coded independently by pairs of trained research assistants to identify cases related to self-harm…[i]nter-anno-tator checks reveal that agreement between research assistants over this period was high (kappa = 0.91).” (p.2). | “This was a retro-spective observational study of all self-harm presentations in persons aged nine years and older” (p.1). | No specific inform-ation reported, how-ever, data for all eligible participants were included in analyses. | NR | “…consensus dis-cussions [were undertaken] with a third member of the research team…to resolve cases [where intent was] unclear” (p.2). | Hospital/medical records used. | No specific inform-ation reported, how-ever, it is likely the same mode of data collection was used for all participants. | The study reported outcome(s) at pre-sentation and/or immediately post-discharge and so it is likely the length of the prevalence period was appro-priate. | There were no errors in the reporting of the numerator(s) and denominator(s) for the prevalence. |
| --- | --- | --- | --- | --- | --- | --- | --- | --- | --- | --- |

**Notes:** NR: not reported.

^1^ The terms ‘non-response bias’ and ‘missing data bias’ are often used interchangeably (e.g., see Richardson HA & Simmering MJ (2020). Missing data in Research. Business and Management, epub ahead of print, DOI: [10.1093/acrefore/9780190224851.013.226](https://doi.org/10.1093/acrefore/9780190224851.013.226).

1. Bayramoglu A, et al. (2015). Demographic and clinical differences of aggressive and non-aggressive suicide attempts in the emergency department in the Eastern Region of Turkey. *Iran Red Crescent Med J*, 17: e24666. [↑](#footnote-ref-1)
2. Al-Thani H, et al. (2020). Traumatic injuries associated with suicide attempts: A retrospective study from single national level 1 trauma center. *Int J Crit Illn Inj Sci*, 10: 92-8. [↑](#footnote-ref-2)
3. Bennewith O, et al. (2004). Variations in the hospital management of self-harm in adults: An observational study. *Br Med J*, 328: 1108-9. [↑](#footnote-ref-3)
4. Bergen H, et al. (2007). Variations in time of hospital presentation for deliberate self-harm and their implications for clinical services. *J Affect Disord*, 98: 227-37. [↑](#footnote-ref-4)
5. Chang S-S, et al. (2015). Self-harm among people of Chinese origin versus White people living in England: A cohort study. *BMC Psychiatry*, 15: 79. [↑](#footnote-ref-5)
6. Clements C, et al. (2020). Exploring characteristics and risk of repetition in people who fail to report previous hospital presentations for self-harm: A case-control study using data from The Manchester Self-Harm Project. *J Affect Disord*, 262: 77-82. [↑](#footnote-ref-6)
7. Diggins E, et al. (2017). Age-related differences in self-harm presentations and subsequent management of adolescents and young adults at the emergency department. *J Affect Disord*, 208: 399-405. [↑](#footnote-ref-7)
8. Gardner KJ, et al. (2020). The significance of site of cut in self-harm in young people. *J Affect Disord*, 266: 603-9. [↑](#footnote-ref-8)
9. Farooq B, Clements C, Hawton K, et al. (2021). Self-harm in children and adolescents by ethnic group: An observational cohort study from the Multicentre Study of Self-Harm in England. *Lancet Child Adolesc*, 5: 782-91. [↑](#footnote-ref-9)
10. Haw C, et al. (2006). Deliberate self-harm patients of no fixed abode: A study of characteristics and subsequent deaths in patients presenting to a general hospital. Soc Psychiatry Psychiatr Epidemiol, 41: 918-25. [↑](#footnote-ref-10)
11. Hawton K, et al. (2021). Self-harm on roads: Register-based study of methods and characteristics of individuals involved. *J Affect Disord*, 282: 46-50. [↑](#footnote-ref-11)
12. Hawton K, et al. (2007). Self-harm in England: A tale of three cities. Multicentre study of self-harm. *Soc Psychiatry Psychiatr Epidemiol*, 42: 513-21. [↑](#footnote-ref-12)
13. Hawton K, et al. (2012). Epidemiology and nature of self-harm in children and adolescents: Findings from the multicentre study of self-harm in England. *Eur Child Adolesc Psychiatry*, 21: 369-77. [↑](#footnote-ref-13)
14. Hiles S, et al. (2015). General hospital-treated self-poisoning in England and Australia: Comparison of presentation rates, clinical characteristics and aftercare based on sentinel unit data. *J Psychosom Res*, 78: 356-62. [↑](#footnote-ref-14)
15. Horrocks J, et al. (2003). Self-injury attendances in the accident and emergency department: Clinical database study. *Br J Psychiatry*, 183: 34-9. [↑](#footnote-ref-15)
16. Kapur N, et al. (2004). Emergency department management and outcome for self-poisoning: A cohort study. *Gen Hosp Psychiatry*, 26: 36-41. [↑](#footnote-ref-16)
17. Kapur N, et al. (2006). The repetition of suicidal behaviour: A multicentre cohort study*. J Clin Psychiatry*, 67: 1599-1609. [↑](#footnote-ref-17)
18. Kapur N, et al. (2013). Does clinical management improve outcomes following self-harm? Results from the Multicente Study of Self-Harm in England. *PLoS One*, 8: e70434. [↑](#footnote-ref-18)
19. Kapur N, et al. (2015). Hospital management of suicidal behaviour and subsequent mortality: A prospective cohort study. *Lancet Psychiatry*, 2: 809-16. [↑](#footnote-ref-19)
20. Lilley et al. (2008). Hospital care and repetition following self-harm: multicentre comparison of self-poisoning and self-injury. *Br J Psychiatry*, 192: 440-5. [↑](#footnote-ref-20)
21. Mahadevan S, et al. (2010). Deliberate self-harm in Oxford University students, 1993-1995: A descriptive and case-control study. *Soc Psychiat Epidemiol*, 45: 211-9. [↑](#footnote-ref-21)
22. Murphy E, et al. (2010). Assessment rates and compliance with assertive follow-up after self-harm: Cohort study. *Arch Suicide Res*, 14: 120-34. [↑](#footnote-ref-22)
23. Mutphy E, et al. (2012). Risk factors for repetition and suicide following self-harm in older adults: multicentre cohort study. *Br J Psychiatry*, 200: 399-404. [↑](#footnote-ref-23)
24. Ness J, et al. (2015). Alcohol use and misuse, self-harm and subsequent mortality: an epidemiological and longitudinal study from the multicentre study of self-harm in England. *Emerg Med J*, 32: 79-9. [↑](#footnote-ref-24)
25. Oude Voshaar RC, et al. (2011). First episode of self-harm in older age: A report from the 10-year prospective Manchester Self-Harm Project. *J Clin Psychiatry*, 72: 737-43. [↑](#footnote-ref-25)
26. Pitman A, et al. (2020). Comparing short-term risk of repeat self-harm after psychosocial assessment of patients who self-harm by psychiatrists or psychiatric nurses in a general hospital: Cohort study*. J Affect Disord*, 272: 158-65. [↑](#footnote-ref-26)
27. Steeg S, et al. (2018). Routine hospital management of self-harm and risk of further self-harm: Propensity score analysis using record-based cohort data. *Psychol Med*, 48: 315-26. [↑](#footnote-ref-27)
28. Steeg S, et al. (2018). Suicide and all-cause mortality following routine hospital management of self-harm: Propensity score analysis using multicentre cohort data. *PLoS One*, 13: e0204670. [↑](#footnote-ref-28)
29. Cooper J, et al. (2013). Are hospital services for self-harm getting better? An observational study examining management, service provision and temporal trends England. *BMJ Open*, 3: e003444. [↑](#footnote-ref-29)
30. Hawton K, et al. (1990). Deliberate self-poisoning and self-injury in older people. *Int J Geriatr Psychiatry*, 5: 367-73. [↑](#footnote-ref-30)
31. Hawton K, et al. (1992). Deliberate self-poisoning and self-injyury in adolescents: A study of characteristics and trends in Oxford, 1976-89. *Br J Psychiatry*, 161: 816-23. [↑](#footnote-ref-31)
32. Hawton K, et al. (1996). Deliberate self-poisoning and self-injury in children and adolescents under 16 years of age in Oxford, 1976-1993. *Br J Psychiatry*, 169: 202-8. [↑](#footnote-ref-32)
33. Hawton K, et al. (2006). Deliberate self-harm in people aged 60 years and over: Characteristics and outcome of a 20-year cohort. *Int J Geriatr Psychiatry*, 21: 572-81. [↑](#footnote-ref-33)
34. Cho M, et al. (2020). Factors associated with psychiatric ward admission in the emergency department after a suicide attempt: The Risk-Rescue Rating Scale (RRRS) and biochemical markers. *Signa Vitae*, 16: 97-104. [↑](#footnote-ref-34)
35. Kapur K, et al. (2002). Effect of general hospital management on repeat episodes of deliberate self poisoning: Cohort study. *BMJ*, 325: 866-7. [↑](#footnote-ref-35)
36. Dani A, Balachandran S, McGill K, et al. (2022). Prevalence of depression and predictors of discharge to a psychiatric hospital in young people with hospital-treated deliberate self-poisoning at an Australian sentinel unit. *Int J Environ Res Pub Health*, 19: 15753. [↑](#footnote-ref-36)
37. McGill K, Whyte IM, Sawyer L, et al. (2022). Effectiveness of the Hunter Way Back Support Service: An historical controlled trial of a brief non-clinical after-care program for hospital-treated deliberate self-poisoning. *Suicide Life Threat Behav*, 52: 500-14. [↑](#footnote-ref-37)
38. Jackson M, et al. (2020). Hospital-treated deliberate self-poisoning in the older adult: Identifying specific clinical assessment needs. *Aust N Z J Psychiatry*, 54: 591-601. [↑](#footnote-ref-38)
39. Bridge JA, et al. (2012). Outpatient care of young people after emergency treatment of deliberate self-harm. *J Am Acad Child Adolesc Psychiatry*, 51: 213-22. [↑](#footnote-ref-39)
40. Olfson M, et al. (2013). Emergency department recognition of mental disorders and short-term outcome of deliberate self-harm. *Am J Psychiatry*, 170: 1442-50. ^*^ Numerator exceeds denominator in the study report. [↑](#footnote-ref-40)
41. Carter GL, et al. (2005). Postcards from the Edge project: Randomised controlled trial of an intervention using postcards to reduce repetition of hospital treated deliberate self poisoning. *BMJ,* 331: 805. [↑](#footnote-ref-41)
42. Carter GL, et al. (2005). Clinical management for hospital-treated deliberate self-poisoning: Comparisons between patients with major depression and borderline personality disorder. *Aust N Z J Psychiatry*, 39: 266-73. [↑](#footnote-ref-42)
43. Carter GL, et al. (2006). Psychiatric hospitalization after deliberate self-poisoning. *Suicide Life Threat Behav*, 36: 213-22. [↑](#footnote-ref-43)
44. Ticehurst S, et al. (2002). Elderly patients with deliberate self-poisoning treated in an Australian general hospital. *Int Psychogeriatr*, 14: 97-105. [↑](#footnote-ref-44)
45. Whyte IM, et al. (1997). A model for the management of self-poisoning. *MJA*, 167: 142-6. [↑](#footnote-ref-45)
46. Suominen KH, et al. (2002). Health care contacts before and after attempted suicide. *Soc Psychiatry Psychiatr Epidemiol*, 37: 89-94. [↑](#footnote-ref-46)
47. Suominen K, et al. (2004). Health care contacts before and after attempted suicide among adolescent and young adult versus older suicide attempters. *Psychol Med*, 34: 313-21. [↑](#footnote-ref-47)
48. Suominen K, et al. (2004). Elderly suicide attempters with depression are often diagnosed only after the attempt. *Int J Geriatr Psychiatry*, 19: 35-40. [↑](#footnote-ref-48)
49. Walkup JT, et al. (2012). A systematic review of validated methods for identifying suicide or suicidal ideation using administrative or claims data. *Pharmacoepidemiol Drug Saf*, 21: 174-82. [↑](#footnote-ref-49)
50. Williams S. (2015). Establishing a self-harm surveillance register to improve care in a general hospital. *Br J Ment Health Nurs*, 4, 20–25. [↑](#footnote-ref-50)
